# Scale-Fractal Detrended Fluctuation Analysis for Fault Diagnosis of a Centrifugal Pump and a Reciprocating Compressor

**DOI:** 10.3390/s24020461

**Published:** 2024-01-11

**Authors:** Ruben Medina, René-Vinicio Sánchez, Diego Cabrera, Mariela Cerrada, Edgar Estupiñan, Wengang Ao, Rafael E. Vásquez

**Affiliations:** 1 CIBYTEL-Engineering School, Universidad de Los Andes, Mérida 5101, Venezuela; 2 GIDTEC, Universidad Politécnica Salesiana, Cuenca 010105, Ecuador; dcabrera@ups.edu.ec (D.C.); mcerrada@ups.edu.ec (M.C.); 3 Mechanical Engineering Department, Universidad de Tarapacá, Arica 1010069, Chile; eestupin@uta.cl; 4 National Research Base of Intelligent Manufacturing Service, Chongqing Technology and Business University, 19# Xuefu Avenue, Nan’an District, Chongqing 400067, China; aowg@ctbu.edu.cn; 5 School of Engineering, Universidad Pontificia Bolivariana, Circular 1 # 70-01, Medellín 050031, Colombia; rafael.vasquez@upb.edu.co

**Keywords:** detrended fluctuation analysis, reciprocating compressors, multi-fault classification, centrifugal pump, multi-fractal feature extraction, vibration signals

## Abstract

Reciprocating compressors and centrifugal pumps are rotating machines used in industry, where fault detection is crucial for avoiding unnecessary and costly downtime. A novel method for fault classification in reciprocating compressors and multi-stage centrifugal pumps is proposed. In the feature extraction stage, raw vibration signals are processed using multi-fractal detrended fluctuation analysis (MFDFA) to extract features indicative of different types of faults. Such MFDFA features enable the training of machine learning models for classifying faults. Several classical machine learning models and a deep learning model corresponding to the convolutional neural network (CNN) are compared with respect to their classification accuracy. The cross-validation results show that all models are highly accurate for classifying the 13 types of faults in the centrifugal pump, the 17 valve faults, and the 13 multi-faults in the reciprocating compressor. The random forest subspace discriminant (RFSD) and the CNN model achieved the best results using MFDFA features calculated with quadratic approximations. The proposed method is a promising approach for fault classification in reciprocating compressors and multi-stage centrifugal pumps.

## 1. Introduction

Early fault detection in rotating machinery prevents unplanned downtime and catastrophic damage. Condition monitoring is a helpful tool that measures various variables, such as vibration, to detect faults early [1,2]. The standard methods used in condition monitoring of rotating machinery include vibration, thermography, oil, current signature, and acoustic sound analysis. The most common method corresponds to vibration analysis, where vibration signals are recorded, and their analysis of time and frequency features are helpful in identifying potential problems in the machinery [3].

In a reciprocating compressor, a piston driven by a motor moves in a cylindrical compression chamber for the operating fluid until attaining the desired operating pressure [4,5,6]. Like any other rotating machine, the compressor has vulnerable parts that can fail, producing dangerous accidents and economic losses. The most common types of failures in reciprocating compressors are piston seal failures, bearing failures, and valve failures. Piston seal failures can allow the compressed fluid to escape, which can cause fires or explosions. Bearing failures can cause the compressor to vibrate excessively, leading to other failures. Similarly, valve failures can prevent the compressor from operating correctly and lead to failures in other system components.

The centrifugal pump is a machine that can transfer kinetic energy to a fluid, increasing its flow velocity [7]. The centrifugal pumps rotate an impeller composed of a set of vanes or blades that exert a centrifugal force on the fluid, making it flow outward from the impeller center. The fluid then passes through the volute, a casing surrounding the impeller, and is discharged from the pump. The performance of centrifugal pumps depends on several factors, such as the size and speed of the impeller, the type of fluid used, and the pressure and flow rate requirements of the application. The construction of centrifugal pumps can be tailored to handle various fluids such as water, oil, gas, and chemicals [8]. Similar to any machine, they are susceptible to severe faults that can affect many industrial processes where they are located. Consequently, condition monitoring is a valuable tool that enables the early detection of faults.

The recorded vibration signals contain signatures representing mechanical faults in centrifugal pumps and reciprocating compressors. Consequently, many data-driven fault diagnosis approaches are based on extracting features from these vibration signals and using such features for fault classification or prognosis through machine learning approaches. However, there is significant ongoing research and development in machine learning, including deep learning. The main challenge in this application is extracting features from the vibration signals that can accurately represent the faults for effective fault detection. One limitation is that fault signatures are often feeble signals, masked by heavy background noise [9]. Most of the time, feature extraction methods require complex preprocessing to improve the signal-to-noise ratio. In addition, centrifugal pumps with impeller faults are non-linear dynamical systems [10], susceptible to chaotic behavior [11,12] and long-term fractal correlations [13]. Consequently, vibration signals in such equipment must be better represented by standard statistical features or even spectral analysis [14,15]. The availability of features that accurately represent fault signatures is crucial for achieving higher-level challenges like predictive and health management (PHM), where condition-based maintenance and predictive maintenance are vital tasks that depend heavily on the accurate data measured from the system [16,17]. Like centrifugal pumps, the research challenges in reciprocating compressors are centered around PHM and require data features that can accurately represent potential faults. In addition, reciprocating compressors are positive displacement compressors commonly used in the petrochemical industry; their work in harsh environments could lead to faults in the main components of the compressor. In addition, recorded signals are usually affected by disturbances and interferences that represent severe challenges to feature extraction technologies [18]. Valves, in particular, are components susceptible to faults, and significant challenges exist [19,20] in understanding the mechanisms leading to faults in such components. Within this framework, condition monitoring using vibration signals and extracting useful features is essential for addressing these problems. Challenges also arise from the non-stationary and non-linear dynamics of these complex mechanical systems [21] as well as the chaotic or long-term fractal correlations [22], which are present in this type of time series.

Early fault diagnosis involves detecting and identifying faults in a system before they cause failure. This detection can be achieved by monitoring the system for signs of degradation or using data-driven techniques to identify patterns indicative of a fault. The early fault diagnosis techniques are usually data-driven technologies, where several signals can be sensed, such as vibration, acoustic emission, sound, flow rate, temperature, pressure, or electric currents. The fault diagnosis can be feasible using valuable features extracted from such signals. The features extracted from recorded signals are fed to machine learning models [23,24,25,26] that perform the automatic detection of faults.

A condition classification system for reciprocating compressors is reported in [27]. This research extracted several statistical features from the discrete wavelet representation of the vibration signal. These features were used to feed a neural network that classified the compressor as healthy or faulty, achieving an accuracy of up to 100%. Although the approach is accurate, it can only distinguish between healthy and faulty states. Concerning machine learning techniques used in various application examples related to fault detection and classification, consider neural networks [27,28,29], the logistic regression algorithm [5], k-Nearest neighbors (k-NN) [30], random forests [31], multi-layer perceptron [17,28], and support vector machines (SVMs) [32,33]. These mentioned approaches are applications for centrifugal pumps or reciprocating compressors. However, some of these applications address the problem of binary fault classification; some focus on classifying a small set of valve faults, others investigate cavitation faults, and some are related to fault prognostics. Overall, previous research shows that there is still room for improvement in feature extraction aimed at fault classification in both types of devices investigated.

A method for fault detection in impellers and bearings of a centrifugal pump was proposed by Hamomd et al. [34]. The modulation signal bispectrum (MSB) method is the methodological tool used for analyzing recorded vibration signals to perform fault detection. The MSB extracts features of modulating components at low-frequency bands that are useful for detecting faults concerning bearing defects and impeller blockages. Only three different conditions were analyzed, corresponding to a healthy state, bearing outer-race defect plus small impeller blockage fault, and bearing outer-race defect plus large impeller blockage fault. In addition, the authors did not perform classifications of these types of faults. Their research is limited to analyzing the vibration signal spectrum under different flow rates and for a specific range of frequencies. In the work by Tan et al. [35], an investigation was conducted on a centrifugal pump with multi-malfunction. This research analyzed a single-suction centrifugal pump with single-stage seal ring abrasion and a broken blade, focusing on pump performance and flow characteristics. The authors recorded the vibration signal, radial force, inner flow, and pressure of a centrifugal pump with multi-faults and compared them to those from a healthy pump. The results from the numerical simulation were also included. The efficiency of the multi-malfunction pump is lower than that of the healthy pump. The vibration signal is affected, and new characteristic frequencies are shown in the signal spectrum. The peak-to-peak pressure signal decreased at the pump outlet and increased at the volute tongue. The research is devoted to detecting the malfunction of the combined fault based on the analysis of the vibration signal spectrum. However, the research does not report any classification of the type of malfunction.

In [36], an application involving multi-fractal detrended fluctuation analysis (DFA) for detecting faults in valves of a reciprocating compressor is reported. During preprocessing, the vibration signal’s empirical wavelet transform (EWT) was combined with state-adaptive morphological filters (SMFs). Then, the modal function was quantitatively analyzed using MFDFA aimed at fault detection. Although the method can identify the fault type in the valve, it depends on a complex preprocessing methodology. A feature extraction method that combines variational mode decomposition (VMD) and MFDFA in a reciprocating compressor is reported in [37]. The vibration signals are decomposed using VMD. Each resultant intrinsic mode function (IMF) is analyzed using MFDFA, and a set of eigenvectors providing higher recognition accuracy is selected using principal component analysis (PCA). The method is evaluated for detecting four types of valve faults in a reciprocating compressor. In [38], multi-fractal spectral parameters of four sensor signals recorded from a centrifugal pump under different operating conditions are extracted as features fed to a backpropagation neural network. Then, the outputs from the neural networks are fused using the Dempster–Shafer evidence theory to attain the final diagnosis. The research concerning DFA, MFDFA, and the combination of EMD with MFDFA shows that these features are sensible to noise in the vibration signals [39]. Their application for fault detection in centrifugal pumps and reciprocating compressors heavily relies on using complex denoising preprocessing methodologies.

Detection of long-range correlations in time series and fractal scaling properties is feasible using the detrended fluctuation analysis (DFA) method [40]. This method has been successfully applied in various fields, including fault detection. Non-linear and non-stationary time series can be analyzed using DFA to quantify a signal’s long-range correlation. Changes in the long-range correlation of a signal, indicative of a fault, can be identified using DFA. An example of applying multi-fractal DFA analysis for fault diagnosis in rotating machinery is reported in [41]. Specifically, in this research, the application is related to the fault diagnosis in gearboxes and roller bearings. The extracted features are proposed using a multi-fractal manifold method to extract the relevant features. The authors show how the vibration signals have relevant multi-fractal characteristics. Additionally, the authors show that multi-fractal DFA can enhance fractal characteristics at different scales, although their approach does not leverage multi-scale estimation. The DFA method first detrends the signal, removing the trend from the signal. The trend is the long-term average of the signal. Once the trend is removed, the DFA method calculates the fluctuation function, which measures the signal variation around the trend. The fluctuation function is estimated for several time scales. Although MFDFA has proven useful for fault detection in reciprocating compressors and centrifugal pumps, the research has primarily focused on the detection and classification of a small set of fault conditions by extracting MFDFA features from preprocessed vibration signals using various denoising methodologies. In addition, the fractal spectrum corresponds to the features commonly used for fault detection. The fractal spectrum and the self-similarity coefficient α have been mainly used for a selected scale and possibly for a small set of multi-fractal parameters *q*.

In this research, we propose the utilization of the self-similarity coefficient α extracted from the raw vibration signal as a bi-dimensional surface estimated for multiple scales and multiple fractal parameter values (*q*). This feature type has been previously used in the biomedical engineering domain for heart rate and blood pressure characterization [42]. However, to our knowledge, this type of feature has not been applied for fault diagnosis in rotating machinery.

The original contributions of this research are as follows:

1. A novel bidimensional feature extracted from the multi-scale, multi-fractal DFA analysis of raw vibration signals was proposed. Such a 2D surface can capture the fault signature’s scale or fractal features from the vibration signals without requiring the application of preprocessing or denoising methodologies.
2. This scale-fractal DFA representation extracted from the raw vibration signals efficiently diagnoses faults in a reciprocating compressor and a centrifugal pump. The performance of this novel feature was evaluated by classifying faults using three different vibration signal datasets: (a) a dataset with 17 multi-valve fault conditions, and (b) a dataset with 13 multi-fault conditions, both acquired from a reciprocating compressor. Additionally, we considered a dataset with 13 fault conditions recorded from a multi-stage centrifugal pump.
3. We propose a 2D robust scale-fractal feature representation extracted from vibration signals that can be easily adapted for fault classification using either deep learning-based or classical machine learning models.

This paper is organized as follows. Section 2 contains the theoretical background regarding feature extraction using MFDFA. Section 3 includes details on the experimental setup, and Section 4 describes the methodology for fault classification using scale-fractal DFA features. Section 5 presents the experimental results, and Section 6 discusses the results. Finally, Section 7 includes a summary of the research presented.

## 2. Theoretical Background

### 2.1. Multi-Fractal Time Series

A mono-fractal time series only exhibits self-similarity over a single scale. This similarity means that the time series has statistical properties that are relatively similar as the time series are divided into smaller segments. In particular, the autocorrelation, variance, and Hurst exponent can be used for describing the mono-fractal time series [13]. In contrast, a multi-fractal time series represents a fractal system generalization that requires other descriptors apart from the fractal dimension to describe its dynamics. Consequently, their description can be performed using the singularity spectrum and the singularity exponent or Hurst exponent [43].

### 2.2. Detrended Fluctuation Analysis

**Figure 1:**
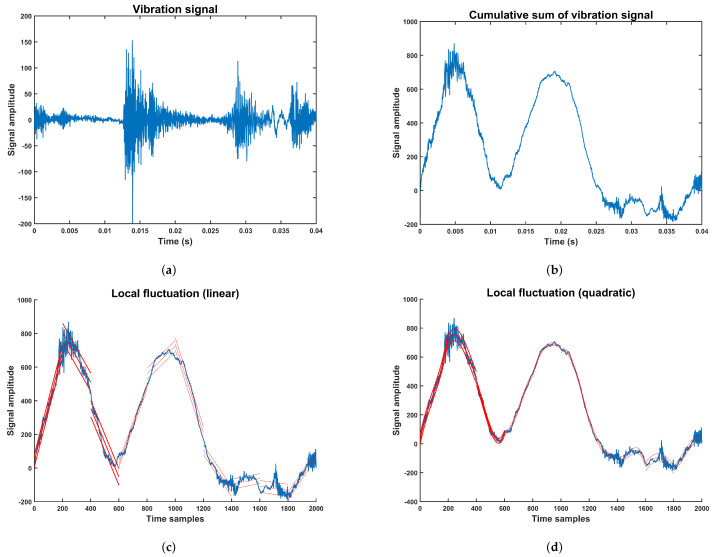
Detrended fluctuation analysis for a vibration signal. The fluctuation signals are represented in red lines. (**a**) Vibration signal from the compressor multi-fault dataset, (**b**) Accumulative vibration signal, (**c**) fluctuation using linear approximation, (**d**) fluctuation using quadratic approximation.

Detrended fluctuation analysis is used to calculate time series self-similarity. DFA enables the estimation of the fluctuation function F(n) for a specific range of time scales *n*, and the slope α for the log−log plot of the fluctuation function [44]. The α slope represents the spectrum of the time series’s local exponents or multi-scale structures. The detrended fluctuation analysis is conducted as follows: given a time series $x_{k}$ with *N* samples, the MFDFA is composed of five steps:

- Step 1: The profile or accumulation sum is determined as [40]:
(1) $$S_{i}=\sum_{k=1}^{i}\left[x_{k}-\bar{x}\right],\;\;\;\;i=1,...,N$$
  where $\bar{x}$ is the mean of the time series $x_{k}$ .
  A vibration signal extracted from the multi-fault dataset is shown in Figure 1a. Their corresponding accumulation sum calculated using Equation (1) is shown in Figure 1b.
- Step 2: The profile accumulation is subdivided into $N_{n}$ segments of equal length *n*, $N_{n}=N/n$ , which are not overlapped. This procedure is applied to the signal in the forward time direction and, starting from the end and going backward, resulting in a total number of segments corresponding to $2N_{n}$ .
- Step 3: The trend is estimated in each $2N_{n}$ segment. This trend calculation is conducted by the least-squares fit of the time series and subsequent estimation of the variance as [40]:
(2) $$F^{2}\left(n,\nu \right)=\frac{1}{n}\sum_{i=1}^{n}{\left\{S\left[\left(\nu -1\right)n+i\right]-s_{\nu }\left(i\right)\right\}}^{2},$$
  for each signal segment ν , $\nu =1,...,N_{n}$ and
(3) $$F^{2}\left(n,\nu \right)=\frac{1}{n}\sum_{i=1}^{n}{\left\{S\left[N-\left(\nu -N_{n}\right)n+i\right]-s_{\nu }\left(i\right)\right\}}^{2},$$
  for $\nu =1,...,2N_{n}$ . In both cases, $s_{\nu }\left(i\right)$ represents a fitting polynomial in segment ν . The possibilities for the polynomial order are linear, quadratic, cubic, or higher-order polynomial. This fitting polynomial leads to different orders of DFA known as DFA1, DFA2, DFA3, … [45]. An example of DFA of order one, or linear DFA, is shown in Figure 1c, and the corresponding DFA of order two, or quadratic DFA, is shown in Figure 1d for a vibration signal.
- Step 4: The fluctuation function of *q* order is the average over all segments, as [40]:
(4) $$F_{q}\left(n\right)={\left\{\frac{1}{2N_{n}}\sum_{\nu =1}^{2N_{n}}{\left[F^{2}\left(n,\nu \right)\right]}^{q/2}\right\}}^{1/q},$$
  where *q* is a real number with values different from zero, if *m* represents the order of DFA, then $F_{q}\left(n\right)$ is defined for n≥m+2 .
- Step 5: The scaling structure of the fluctuation function can be estimated from the log-log plots of $F_{q}\left(n\right)$ as a function of *n* considering several values of *q*. In time series long-range power-law correlated, when *n* has a large value, $F_{q}\left(n\right)$ increases as a power-law [46]:
(5) $$F_{q}\left(n\right)∼n^{h(q)},$$
  where the generalized Hurst exponent h(q) corresponds to the F(n) slope in the log–log plot, usually denoted as *H*.
- Step 6: The time series multi-fractal spectrum can also be calculated as [47]:
(6) $$D\left(q\right)=q\tau^{\prime }\left(q\right)-\tau \left(q\right),$$
  where τ(q)=qh(q)−1 .

Both features H(q) and D(q) are useful for fault detection in rotating machines. However, in this research, we propose using the derivative of the fluctuation function, denoted as α(q,n) because their representation as a 2D function is amenable for processing with CNN models. Also, it is a compact representation of the fault signature of the vibration signal.

### 2.3. Multi-Fractal Detrended Fluctuation Analysis

The time series sample values can result in probability density functions related to multifractality. However, such multifractality can also be present due to long-range fluctuation correlations in the time series [40].

**Figure 2:**
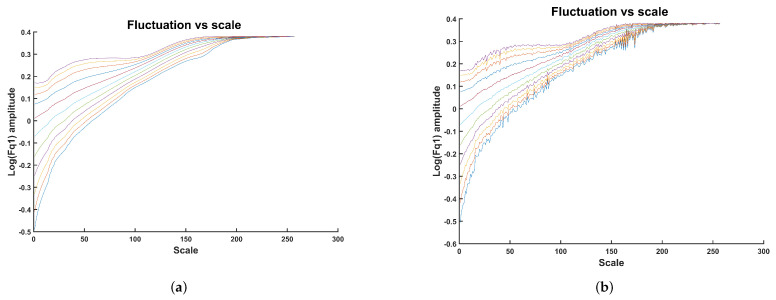
Calculation of $F_{q}\left(n\right)$ and the effect of using maximum overlapped boxes. The parameter *q* was extracted from integers between −5 and +5. Each of the curves plotted in colors represents a *q* value in the mentioned range. (**a**) Maximum overlapping between boxes, (**b**) no overlapping between boxes.

DFA enables the estimation of the α slope in F(n) , representing the fluctuation function, where *n* corresponds to a range of time scales. In particular, α represents the slope of the least-square regression line fitting logF(n) vs. logn . Multi-fractal approaches calculate the fluctuation function considering a positive and negative range of *q*, including q=2 . In this way, positive *q* values amplify the contributions of larger amplitude fractal components, while negative *q* values emphasize smaller amplitude fractal components. This procedure leads to calculating the slope as a function α(q,n) , also known as the self-similarity coefficient. In [44], the calculation of α using the first derivative has been attempted. However, minimizing the noisy variability of F(n) is necessary. This minimization implies estimating F(n) in maximally overlapped blocks of size *n* that are overlapped n−1 samples. An example of a fluctuation calculation using non-overlapped and overlapped windows is shown in Figure 2. The fluctuation was calculated for a range of integers *q* between −5 and +5. A smooth fluctuation representation is obtained with maximally overlapped windows as shown in Figure 2a. In contrast, a noisy representation of the fluctuation is obtained using non-overlapped windows, as shown in Figure 2b. The limitation of using maximally overlapped windows is the high computational cost, making this approach infeasible for long time series. However, in [44], an efficient algorithm for solving this problem is proposed. Such an algorithm is briefly presented in the following paragraph.

### 2.4. Algorithm for Fast Calculation of $F_{q}\left(n\right)$

The example shown in Figure 2 shows two methods of calculating $F_{q}\left(n\right)$ with and without maximum overlapping of calculation blocks. In the second method, the variability of $F_{q}\left(n\right)$ at larger scales could make interpreting results difficult. This variability is highly reduced by using maximum overlapping blocks; however, the computational cost increases, particularly for long time series.

$F_{q}\left(n\right)$ is calculated by Equation (1), where $S_{i}$ is subdivided into *M* blocks. Each has size *n* for a time series of length *N*. We can denote *L*, the quantity of overlapping between contiguous blocks ( 0≤L≤n ). This parameter can be estimated as [44]:

(7) $$M=\left\lfloor \frac{N-n}{n-L}\right\rfloor +1,$$

when we have L=n−1 , all *N* samples are incorporated in the $F_{q}\left(n\right)$ calculation, and we have maximum overlapping. When M=N−n+1 is considered, we have the maximum number of blocks. Each block is detrended using a least-square polynomial fitting, denoted as r(i) . The variance of residual in the detrended block *k* can be expressed as

(8) $$\sigma_{n}^{2}\left(k\right)=\frac{1}{n}\sum_{i=I_{k}}^{I_{k}+n-1}{\left(S_{i}-r\left(i\right)\right)}^{2}$$

where index $I_{k}=\left(n-L\right)\left(k-1\right)+1$ represents the samples included in the *k* block. According to [40], the variability function for DFA is calculated as

(9) $$F_{q}\left(n\right)={\left(\frac{1}{M}\sum_{k=1}^{M}{\left(\sigma_{n}^{2}\left(k\right)\right)}^{q/2}\right)}^{1/q},$$

that is valid for q≠0 , and

(10) $$F_{q}\left(n\right)=e^{\frac{1}{2M}\sum_{k=1}^{M}ln\left(\sigma_{n}^{2}\left(k\right)\right)}$$

is used for calculation when q=0 . Evaluation of $F_{q}\left(n\right)$ has a computational cost mainly related to calculating Equation (8) that involves the calculation of least-square polynomial fitting and the variance for *n* points. As these calculations are repeated for the *M* blocks, and *M* could be close to *N*, the computational cost increases heavily for maximum overlapped blocks. An algorithm for the fast calculation of order 1 and 2 detrending polynomials, denoted as DFA1 and DFA2, has been proposed in [48]. This fast algorithm also includes improvements in evaluating residuals in Equation (8).

## 3. Centrifugal Pump and Reciprocating Compressor Datasets

The environmental conditions of the laboratory are carefully set and kept during the signal acquisition experiment to guarantee that the process is reproducible. The verification of such environmental conditions must be carried out before starting the signal acquisition and during the acquisition process of the data. The values of the environmental parameters that serve as reference for the operation of the centrifugal pump and the reciprocating compressor under normal operating conditions are presented in Table A1. If these values are not within the established range, the data acquisition is invalid. Therefore, the test will be repeated when the environmental conditions are within the specified ranges.

### 3.1. Centrifugal Pump Dataset

**Figure A1:**
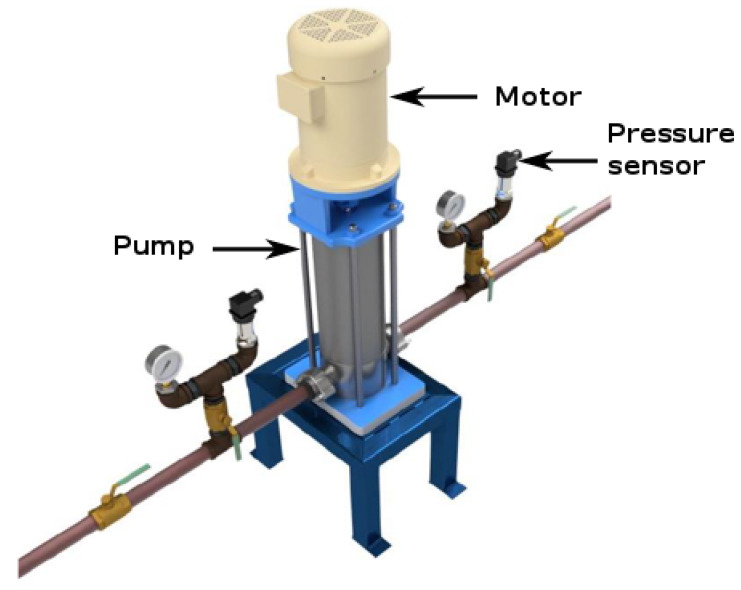
Main parts of the vertical centrifugal pump test bench.

**Figure A2:**
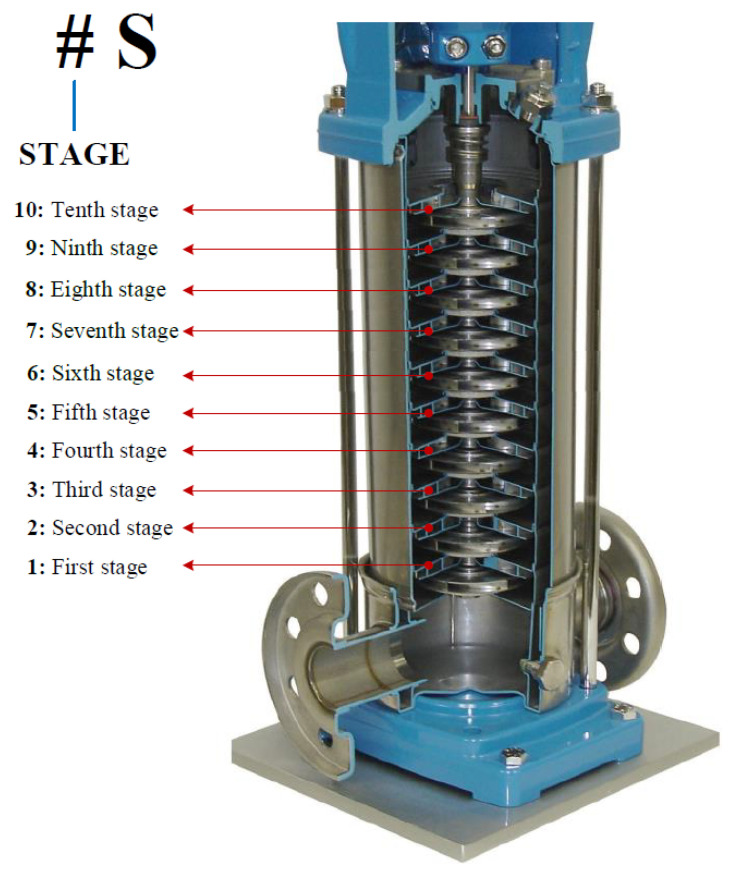
The ten stages of the vertical centrifugal pump. The centrifugal pump model is 3SV10GE4F20 and it is driven by a two-hp induction motor.

The experiments were performed using a vertical ten-stage centrifugal pump model 3SV10GE4F20 driven by a two-hp induction motor that provides rotatory motion at 3500 rpm. Figure A1 shows the vertical centrifugal pump’s main parts. The centrifugal pump’s internal structure with ten stages is shown in Figure A2.

**Figure 3:**
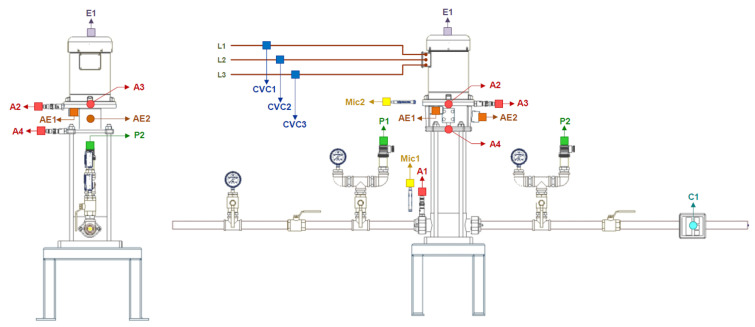
Sensor location for the centrifugal pump. The accelerometer sensors are *A1*, *A2*, *A3*, and *A4*. The sound signals are recorded with microphones Mic1 and *Mic2*. The sensors for acoustic emission signals are *AE1* and *AE2*. The current sensors are *CV1*, *CV2*, and *CV3*. The sensor for rotational motion is a rotary encoder *E1* and *C1* is a flow meter.

The sensor’s location for the centrifugal pump is shown in Figure 3. Several types of sensors are located for monitoring the centrifugal pump. Four accelerometers corresponding to *A1*, *A2*, *A3*, and *A4* are used for measuring the vibration signals. The sound signals are recorded using two acoustic microphones *Mic1* and *Mic2*. The acoustic emission signals are recorded with sensors *AE1* and *AE2*. A rotary encoder *E1* is used for monitoring the rotational motion of the motor. In addition, three current sensors are used, corresponding to *CV1*, *CV2*, and *CV3*. Concerning the accelerometers, sensors *A2* and *A4*—located at the end of the coupling between the centrifugal pump and the motor—have shown excellent classification results in previous research [49]. For this reason, we have chosen sensor *A4* in the research reported in this document. The hardware for vibration signal acquisition is intended for off-line signal processing. The accelerometer, PCB 603C01, is used in the centrifugal pump to acquire the vibration signal. The analog vibration signal is acquired using a National Instruments NI9234 card attached to the NI9188 chassis to stream the digital signal to a laptop computer through a 100 Mbps Ethernet link. The selected sensor *A4* is placed horizontally in the centrifugal pump.

The investigated faults in the centrifugal pump were implemented in the test rig by creating seeded faults in the centrifugal pump impellers. These seeded faults are attained by modifying the physical structure of a faultless component. Research on centrifugal pumps for fault diagnosing using seeded faults has been previously reported in the literature [50,51].

**Figure A3:**
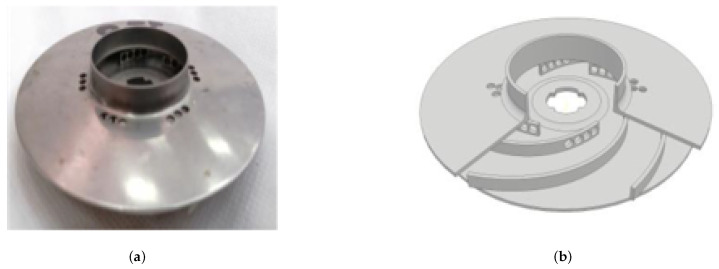
An example of pitting at the entrance of the impeller blades is shown. In addition, up to eight levels of pitting severity can be configured by creating holes in the blades using EDM. (**a**) Holes created using EDM. The diameter of each hole is 2.5 mm and the number of holes varies according to the level of severity for each fault. (**b**) Internal view of the holes created in the impellers.

**Figure A4:**
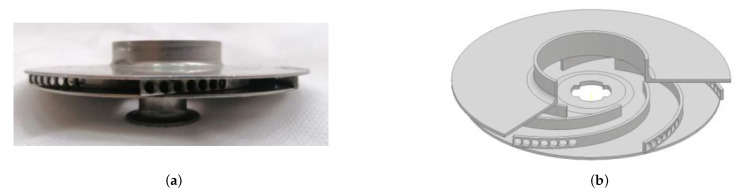
A configuration of pitting at the output of the impeller blades is shown. The configuration of the levels of pitting severity is implemented by creating holes with a diameter size and a number that is increased according to the severity level. (**a**) View of the holes in the output of the impeller blades. (**b**) Internal view of the holes located at the output of the impeller blades.

**Figure A5:**
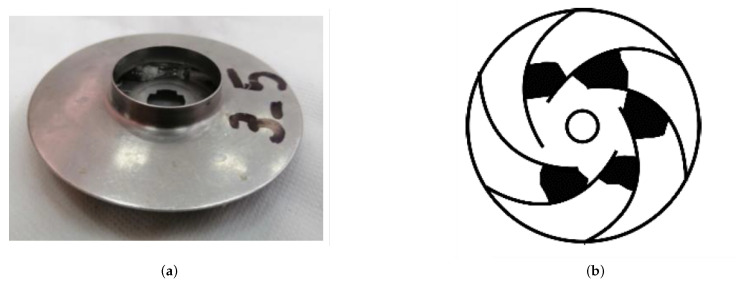
An example of impeller channel blockage is shown. The levels of blockage severity are attained by closing different channels of the impeller. (**a**) Appearance of the impeller channel blockage. (**b**) Drawing representing the impeller channel blockage.

**Figure A6:**
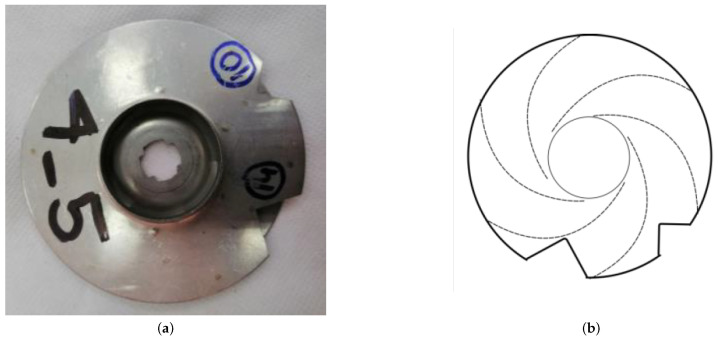
An example of impeller imbalance is shown. The six levels of severity for the impeller imbalance fault are attained by cutting portions of the impeller with increasing area. (**a**) Appearance of the impeller imbalance. (**b**) Drawing representing the impeller imbalance.

The experimental test bench for the centrifugal pump can be configured with several impeller faults. In addition, each impeller fault can be configured with eight or six levels of severity. In particular, the dataset includes five types of conditions: healthy condition (HTH), pitting at the entrance of the impeller blades (PEB) (see Figure A3), pitting at the output of the impeller blades (POB) (see Figure A4), impeller channel blockage (ICB) (see Figure A5), and imbalance impeller (IB) (see Figure A6). In faults, PEB, and POB, the eight levels of pitting severity are attained by creating holes in the blades using electrical discharge machining (EDM) with a diameter size and an increased number of holes according to the severity level. Similarly, six levels of severity for the blockage fault (ICB) are attained by closing the different channels of the impeller. The six levels of severity for the impeller imbalance fault are achieved by cutting portions of the impeller with increasing area.

The fault conditions in the multi-stage centrifugal pump correspond to 13 conditions, labeled P1–P13. Each condition represents one of five conditions previously described for the impellers. The severity of each faulty condition increases according to the stage and the type of condition. Table 1 presents the combination of different faults. The numbers in the columns for stages 5 to 10 represent the severity of the faulty condition presented in the second column.

Six experimental conditions were considered for acquiring the signal dataset. These conditions were related to adjusting the pressure in the discharge valve to the values specified in Table A2. Considering the healthy condition as the baseline, the discharge valve opening was regulated to achieve the specified discharge pressure. The signal acquisition begins by setting an experimental condition Ci with I∈{1,2,...,6} , and ten repetitions of signal acquisition are performed for each fault condition P1 to P13. This process involves the acquisition of 60 vibration signals for each fault condition and a total of 780 vibration signals for the dataset, considering the 13 fault conditions.

Each recorded vibration signal was acquired using a sampling frequency of 50 kHz. Each signal is acquired with a duration of 10 seconds.

### 3.2. Reciprocating Compressor Dataset

**Figure 4:**
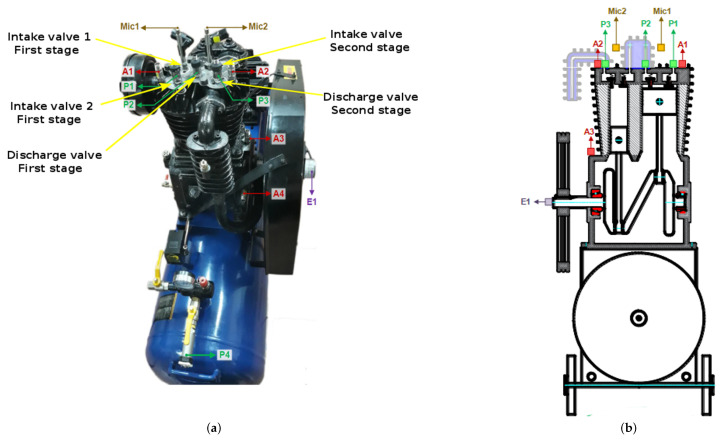
Sensor location for the reciprocating compressor. (**a**) Actual view of the sensor’s location. (**b**) Schema showing the sensor’s location for the reciprocating compressor. The vibration signal is recorded with accelerometers denoted *A1*, *A2*, *A3*, and *A4*.

**Figure A7:**
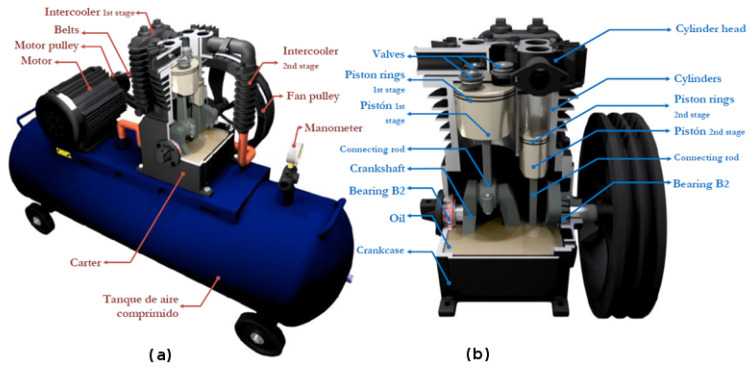
Main parts of the EBG-250 compressor. (**a**) External parts. (**b**) Details of the internal parts.

**Figure A8:**
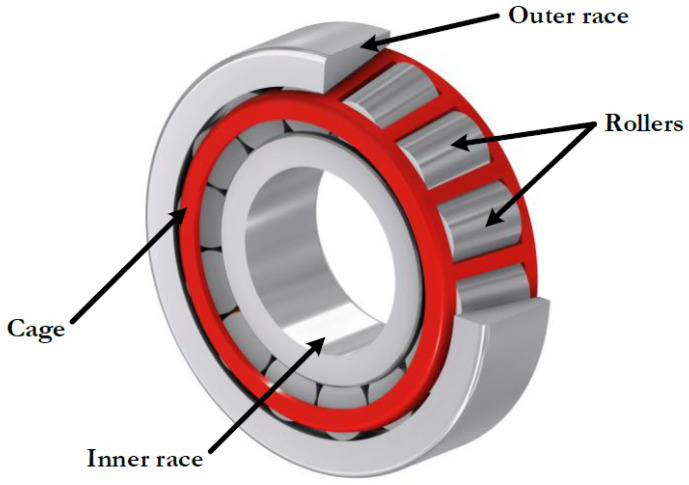
Parts of the tapered roller bearing used in experimentation.

**Figure A9:**
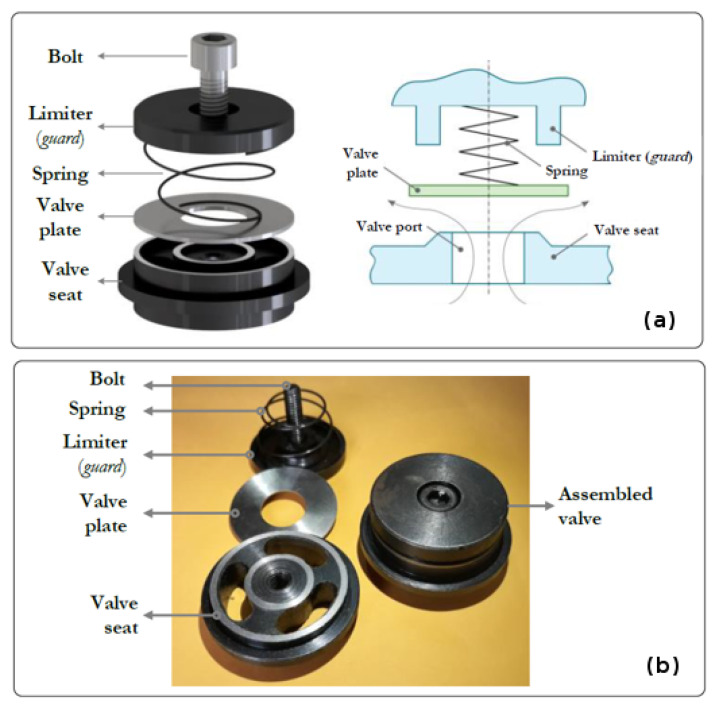
Reciprocating compressor valve assembly and details about their components. (**a**) Components of the valve; (**b**) valve assembly scheme.

The experiments used a two-stage compressor, where rotatory motion at 3470 rpm could be attained with a 5.5 hp induction motor. Two V-belts enabled the transmission of mechanical motion to the compression chamber. In the first stage, two valves, the intake valve (IV), and the discharge valve (DV), enabled the gas transfer to the second stage. The second stage also had two valves, the IV and the DV, enabling air transfer to the tank. The main parts of the compressor are shown in Figure A7. The essential parts are the roller bearings (shown in Figure A8) and the valves (shown in Figure A9). Figure 4 shows the sensor’s location. The view of the sensor’s location in the reciprocating compressor is shown in Figure 4a. Several types of sensors are considered; the accelerometers are labeled as *A1*, *A2*, *A3*, and *A4*. Two acoustic microphones, *Mic1* and *Mic2*, are used for recording the sound. In addition, a rotary encoder *E1* is used. A scheme showing the sensor location concerning the internal components of the reciprocating compressor is shown in Figure 4b. Concerning the accelerometer sensors, previous research using this test rig [49] has shown that sensor *A1*, which is located close to discharge valve 1 of the first stage, provides excellent results concerning the classification of the valve faults. For this reason, we chose accelerometer *A1* in this research. This accelerometer measures the vibration in the vertical direction of the reciprocating compressor. The hardware used for digitally acquiring the vibration signal is similar to that used in the centrifugal pump. The accelerometer sensor is the PCB 603C01, which is connected to the NI9234 card attached to the NI9188 chassis for transferring the digital signal to a laptop computer.

The reciprocating compressor has two roller bearings. The larger bearing has an outside diameter of 80 mm with a bore diameter of 40 mm. The bearing includes 17 rollers, and the width is 24.75 mm. The seat of the valves is made of carbon steel with an external diameter of 41.2 mm and a height of 12.5 mm. The external diameter of the valve plate is 34.5 mm, with a thickness of 1.05 mm and an internal diameter of 14 mm. Such a valve plate is made of stainless steel. A wire made of carbon steel with a diameter of 0.7 mm corresponds to the helical spring. Such a helical spring has a smaller diameter of 20 mm and a larger diameter of 32 mm. The dimensions of the limiter or guard have a height of 5 mm and an external diameter of 34.5 mm. Such a limiter or guard is made of carbon steel. An M6×40 mm bolt attaches all the valve parts.

#### Multi-Valve Fault Dataset

The valve faults investigated in the reciprocating compressor were simulated by taking a faultless component and modifying its physical structure to create the seed of a fault in a controlled environment. This approach has been reported in the literature and used by several authors [5,52,53].

**Figure A10:**
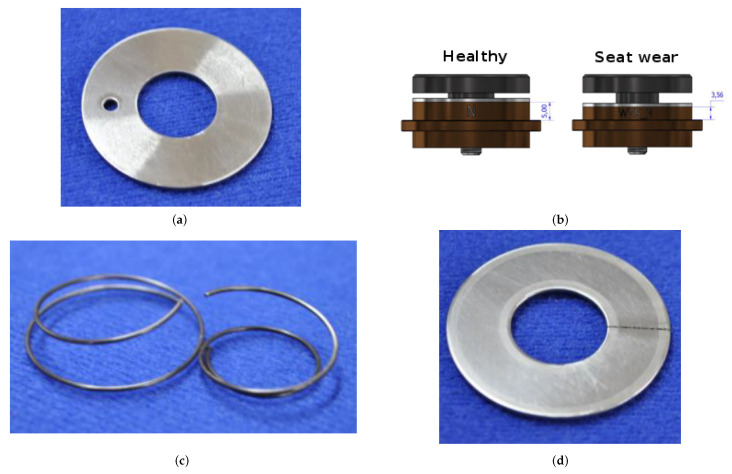
Valve faults in the reciprocating compressor. (**a**) Valve corrosion, (**b**) valve seat wear, (**c**) broken valve spring, and (**d**) valve plate fracture.

The experimental testbed enables the configuration of several valve faults. The valves were configured considering four fault types: (1) wear in the valve seat (VSW), (2) valve plate corrosion (VPC), (3) valve plate fracture (VPF), and (4) broken spring (BS). The configuration of such faults was attained by creating wear in the valve. For instance, the valve seat was modified to reduce its depth from 5.0 mm to 3.56 mm. A hole with a diameter of 2.5 mm was created to simulate the valve plate corrosion. The fracture of the valve plate was manufactured by cutting such a plate with a 1.6 mm diameter thread. Similarly, the broken string was attained by cutting the spring using a 1.6 mm diameter thread. The valve faults are shown in Figure A10.

Seventeen conditions for the valves are configured. Such conditions are denoted as S1 to S17, where the healthy condition is S1, and the rest are faulty. The complete list is reported in Table 2.

During the vibration signal acquisition, a constant motor rotation speed of 57.7 Hz was maintained to achieve a crankshaft rotation frequency of 12.8 Hz. Two crankshaft cycles correspond to one compression cycle. Consequently, it takes approximately 0.156 s to complete a compression cycle. The pressure in the tank was 3 bar and kept constant. Each machinery condition was represented by 15 vibration signals acquired with the sensor. The dataset comprises 255 signals, each sampled at 50 kHz and with a duration of 10 s.

### 3.3. Multi-Fault Dataset

The multi-fault dataset for the reciprocating compressor was created by implementing seeded faults combined in the discharge valve of the second stage and a roller bearing. Seeded faults are frequently used for research in fault detection in reciprocating compressors, either for faults in valves or faults in bearings [54,55].

**Figure A11:**
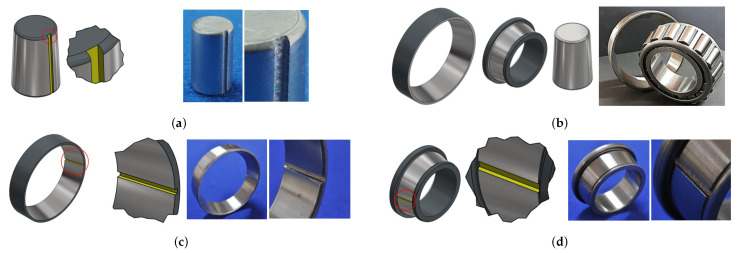
Roller bearing faults in the reciprocating compressor. (**a**) Ball fault, (**b**) healthy roller bearing, (**c**) outer race fault, and (**d**) inner race fault.

The faulty roller bearing, denoted as B1, is located in the second stage’s discharge valve (DV) near the fan pulley. The faulty roller bearing exhibits the following fault types: (a) roller element crack (REC), (b) outer race crack (ORC), and (c) inner race crack (IRC). Both the IRC and ORC were created using the electrical discharge machining (EDM) method, and they are aligned with the rotation axis, covering the entire raceway. The dimensions of these cracks are a depth of 1.0 mm and a width of 2.0 mm. The REC was also created using EDM, and is also aligned with the rotation axis. The dimensions for the REC fault are a depth of 0.5 mm and a width of 1.0 mm. The roller bearing faults are shown in Figure A11.

Four types of faults were configured in the valves: (1) broken spring, (2) valve plate fracture, (3) valve plate corrosion, and 4) wear in the valve seat. The valve seat was reduced in depth by 1.44 mm using EDM to create the valve seat wear. The valve plate corrosion was created with a 2.5 mm diameter hole. A plate cut was used to create the fracture of the valve plate. In this case, the diameter of the thread used for cutting was 1.6 mm. A thread of 1.6 mm in diameter was used to create the spring brake fault by breaking such a spring.

The multi-fault classes were composed of 13 different conditions. The healthy class is denoted as F1. The rest of the classes are denoted as F2–F13.

The different fault classes and the corresponding combinations are presented in Table 3.

The signal acquisition was conducted at a constant motor speed (57.7 Hz) and crankshaft rotation speed (12.8 Hz). A total of 15 vibration signals were acquired from each sensor for each machinery condition. The multi-fault dataset includes 195 vibration signals, digitally acquired at a sampling rate of 50 kHz over 10 s. Concerning the reciprocating compressor, the temperature and pressure values must be monitored during data acquisition and kept within the specified ranges. The temperature and pressure values of the compressor during acquisition are presented in Table A3.

## 4. Methodology

### 4.1. Feature Extraction from the Vibration Signals

**Figure 5:**
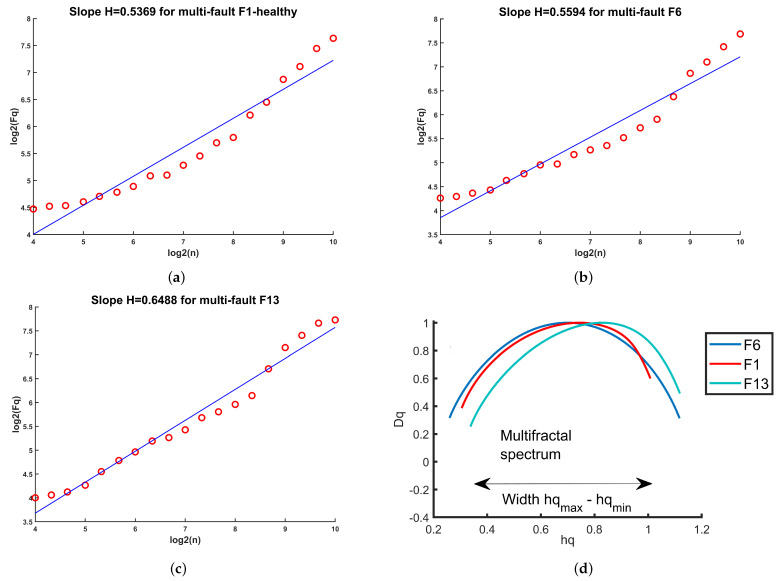
Features from detrended fluctuation analysis for a vibration signal. (**a**) Hurst exponent for a signal from the compressor multi-fault F1 (healthy), (**b**) Hurst exponent for a signal from the compressor multi-fault F6, (**c**) Hurst exponent for a signal from the compressor multi-fault F13, (**d**) fractal spectrum for faults F1, F6, and F13.

The DFA features commonly reported in the literature concerning diagnosing faults in rotating machinery are the Hurst exponent and the fractal spectrum, calculated from $F_{q}\left(n\right)$ . Both features are shown in Figure 5 for several fault conditions in the reciprocating compressor. Even when several features extracted from the fluctuation function have been used for fault detection in rotating machinery, in this research, we selected the derivative of the fluctuation function because this feature implicitly contains information about the fault signature that other derived features try to recover. In addition, feature α(q,n) is a bi-dimensional array that can be easily converted to images for classification using convolutional neural networks. The feature extraction was performed using a similar procedure in the three vibration signal datasets. Six non-contiguous fragments of 32,768-time samples were selected in each vibration signal. The MFDFA was performed for each signal fragment to obtain the fluctuation $F_{q}\left(n\right)$ and the fluctuation function α(q,n) derivative. The parameter *q* varied between −5 and +5 as q=[−5,−4,−3,−2,−1,0,+1,+2,+3,+4,+5] . The algorithms were applied, considering the maximum overlapping of contiguous blocks, using the algorithm described in [48]. The calculated fluctuation function $F_{q}\left(n\right)=F\left(q,n\right)$ is an array of size 11×262 . The multi-fractal DFA coefficients α(q,n) are obtained as this array’s first derivative of $logF_{q}\left(n\right)\;vs\;logn$ . Both results for DFA1 and DFA2 are obtained.

**Figure 6:**
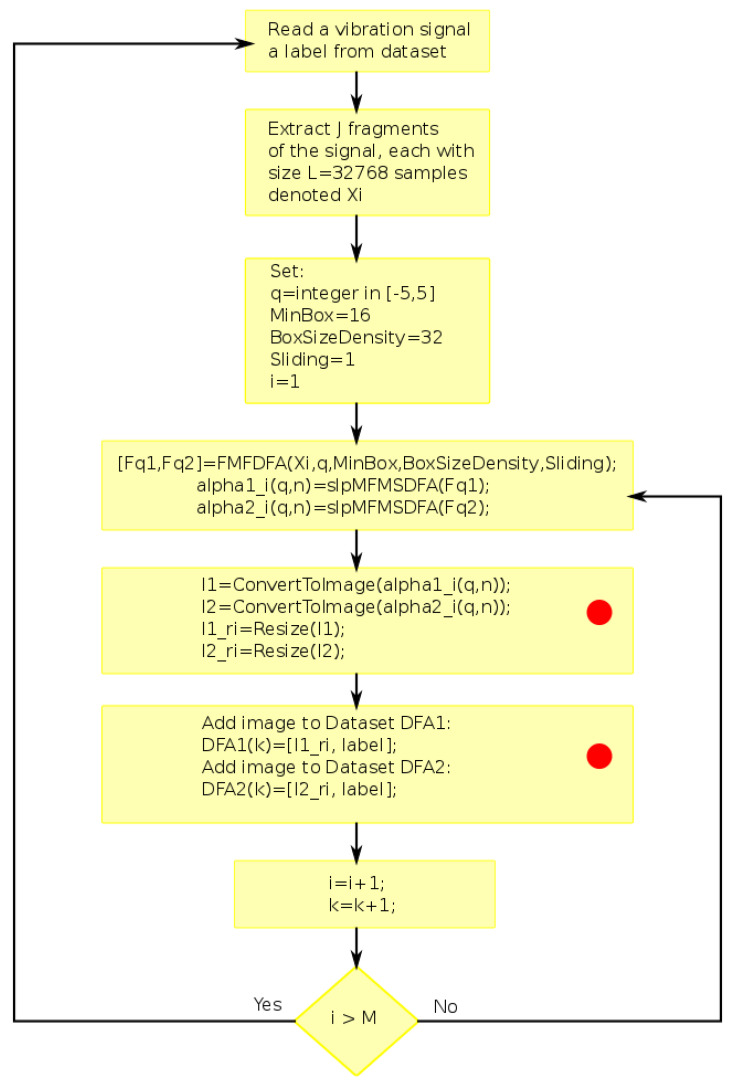
Feature extraction method. The process starts with k=1 and runs until all files in the vibration signal dataset have been processed. A set of J=6 fragments is extracted from each signal. The steps labeled with a red dot are modified when the features are intended for classification using classical machine learning models. In that case, the arrays $\alpha_{1}\left(q,n\right)$ and $\alpha_{2}\left(q,n\right)$ are not converted to images but are resampled to obtain two arrays of size 11×16 . Each array is reshaped as a vector and concatenated with the corresponding label to obtain the feature dataset DFA1 (or DFA2).

The procedure for extracting the features from the vibration signal datasets is presented in Figure 6. The procedure uses the methodology described in [48]. Two Matlab functions distributed as supplementary material of the cited research are used. The first function is *FMFDFA(·)*, which is used for estimating the fluctuations $F_{1}\left(q,n\right)$ = Fq1 and $F_{2}\left(q,n\right)$ = Fq2 with linear and quadratic approximations. This function is fed with a vector array *Xi* with the signal fragment. It requires the fractal parameter *q*, the minimum scale given by *MinBox*, a parameter defining the logarithmic density of scales *BoxSizeDensity*, and the parameter *Sliding*, which indicates boxes overlapping when set to 1. The second function is *slpMFMSDFA(·)*, which is used for calculating the fractal-scale representation $\alpha_{1}\left(q,n\right)$ = alpha1i(q,n) (or $\alpha_{2}\left(q,n\right)$ = alpha2i(q,n) ) from the corresponding fluctuation and the array of logarithmic scales. The stages labeled with a red dot differ depending on the type of classifier used. In the case of using a CNN classifier, both arrays $\alpha_{1}\left(q,n\right)$ and $\alpha_{2}\left(q,n\right)$ are converted to grayscale images with pixels scaled in the range of [0, 1]; both images are then resized using bicubic interpolation to obtain two images of size 128×128 . The next step corresponds to appending such images and their label to the corresponding feature dataset, denoted as DFA1 or DFA2. When the features are used as input to classical machine learning models, the fractal-scale representations $\alpha_{1}\left(q,n\right)$ and $\alpha_{2}\left(q,n\right)$ are resampled to a size of 11×16 and reshaped as a vector, which is concatenated with the corresponding label and appended to the corresponding feature dataset DFA1 or DFA2.

### 4.2. Fault Classification Using Classical Machine Learning Models

The classification accuracy provided by the extracted features was validated using a set of classical machine learning models, including random forests (RFs) [56], particularly random forest models with subspace discriminant learners (RFSDs), random forest subspaces with nearest neighbors (RFKNN), support vector machines (SVMs) with Gaussian kernel (GSVM) [57], k-nearest neighbor (kNN) with a distance weighting function (wKNN) [58], and neural networks (NNs) [59]. Validation is performed using one dataset for the centrifugal pump (faults described in Table 1) and two datasets for the reciprocating compressor (faults in Table 2 and Table 3). The DFA1 and DFA2 feature sets for each vibration signal dataset were validated regarding their classification accuracy. Since both DFA1 and DFA2 feature arrays have a size of 11×262 , which is too large for input into classical machine learning models, these arrays were resampled to achieve a more manageable size of 11×16 , which is fed to the classical machine learning models. Each of the six feature arrays obtained from each signal is considered a separate feature example, and the label associated with the signal used for its calculation is assigned.

A 10-fold cross-validation was performed to assess each machine learning model’s performance. As the feature dataset was split randomly into a training set (85%) and a test set (15%), the training set was used for cross-validation. The trained cross-validated partitioned model was analyzed using the test set for classification accuracy. This validation procedure was repeated ten times, and the averaged confusion matrix was recorded as the final result for each classification model. Several metrics [60] were used to evaluate each classification model. A detailed description of such metrics is reported in [61]. The confusion matrix and the receiver operator curve (ROC) enable the evaluation of the classifier performance metrics.

### 4.3. Fault Classification Using Convolutional Neural Network Models

Classification of faults using CNN requires the features to be represented as an image. In this research, the selected features corresponding to α(q,n) which, in practice, is an array of size 11×262 , is converted to a gray-scale image. The conversion is performed by resampling the feature array as an array of size 128×128 followed by a rescaling operation to obtain pixels represented in the interval [0,1] . As the features α(q,n) are calculated for six fragments of the vibration signal, each of the six images obtained from each signal is considered an example and is assigned the label associated with the signal used for its calculation.

**Figure 7:**
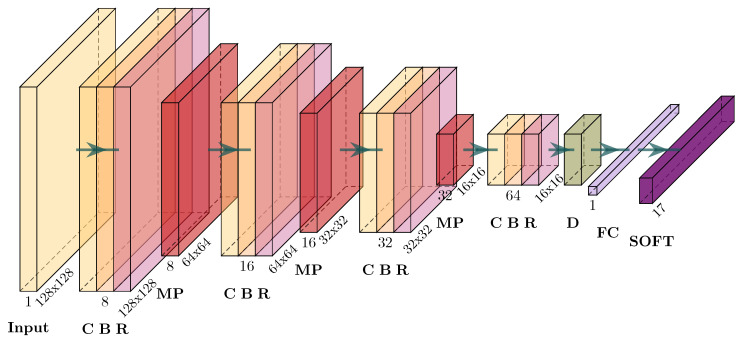
Convolutional neural network architecture. This CNN architecture was used for classifying the faults in the reciprocating compressor and the centrifugal pump. The only modification in each case corresponds to the softmax layer that should be set according to the number of faults to classify.

A relatively simple architecture is used for fault classification. The detailed architecture is shown in Figure 7. The architecture comprises four CBR blocks, each including a convolutional layer, a batch normalization layer, and a ReLU layer, followed by a max-pooling (MP) layer. After the four CBR-MP blocks, a dropout layer (D) is used with a probability of 0.75. This dropout layer is followed by a fully connected layer (FC) and a softmax layer (SOFT) for achieving the required fault classification. All four convolutional layers use a unique filter size of 11×11 . The same architecture was used to classify faults in both the centrifugal pump and the reciprocating compressor, except for the classification layer, which is set according to the number of faults considered. The architecture and parameters were selected after several tests evaluating classification accuracy.

Three CNN models were validated: one for the centrifugal pump and two for the reciprocating compressor. Each model was validated using a 5-fold cross-validation, and the performance metrics were evaluated using the average confusion matrix obtained during cross-validation. Each model was trained using a batch size of 128 and a learning rate of 0.0082 with the Adam solver during 300 epochs.

## 5. Results

**Figure 8:**
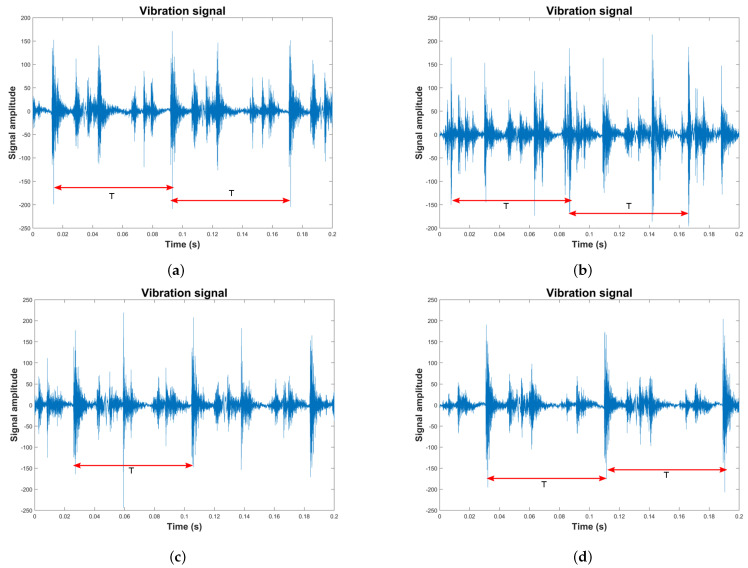
An example of vibration signals extracted from the multi-fault dataset included in the reciprocating compressor is shown. The periodic events are plotted using an arrow line in red and T is the time period of each event in the Figure. (**a**) A representative class F1 signal, (**b**) signal extracted from the class F6 set, (**c**) vibration signal representing the class F10, (**d**) vibration signal representing the class F13.

We begin by illustrating the application of the MFDFA to the reciprocating compressor vibration signal dataset. Several typical vibration signals are shown in Figure 8. The four vibration signals are extracted from the multi-fault dataset. Such signals correspond to the classes F1, F6, F10, and F13. There are differences in appearance for the signals shown. Figure 8a shows the vibration signal corresponding to the healthy class. A periodic event is identified in the signal marked by the roller element, and several events in each period are defined by the valves opening and closing. Figure 8b shows the vibration signal for a roller element crack–valve seat wear combination. In this case, the events corresponding to the roller element are notably altered, mainly dispersed due to the crack. In addition, differences in the events related to the valves are present as changes in amplitude. Figure 8c,d show vibration signals for faults F10 and F13, both with outer race crack. The first had valve seat wear, and the second had a broken spring. While the events marked by the roller elements are similar, the outer race crack and the valve faults modify the rest of the signal events shown in both figures. Even when there are some differences in the signals, performing a quantitative analysis of such signals is necessary for accurate fault classification.

**Figure 9:**
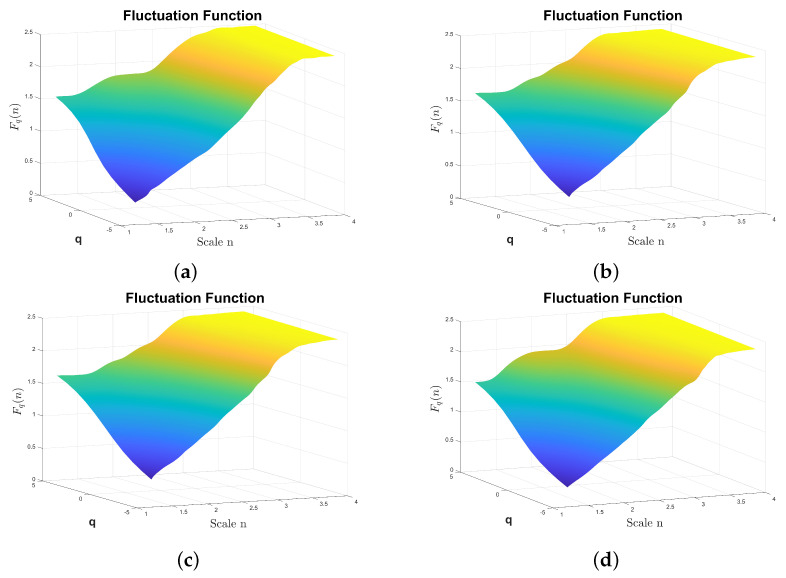
Fq(n) from the reciprocating compressor’s multi-fault dataset calculated by DFA1. The fluctuation amplitude is color coded, the highest amplitudes are plotted using light yellow, the lower amplitudes are plotted in dark blue. (**a**) Fq(n) from the class F1, (**b**) Fq(n) from the class F6, (**c**) Fq(n) from the class F10, (**d**) Fq(n) from the class F13.

**Figure 10:**
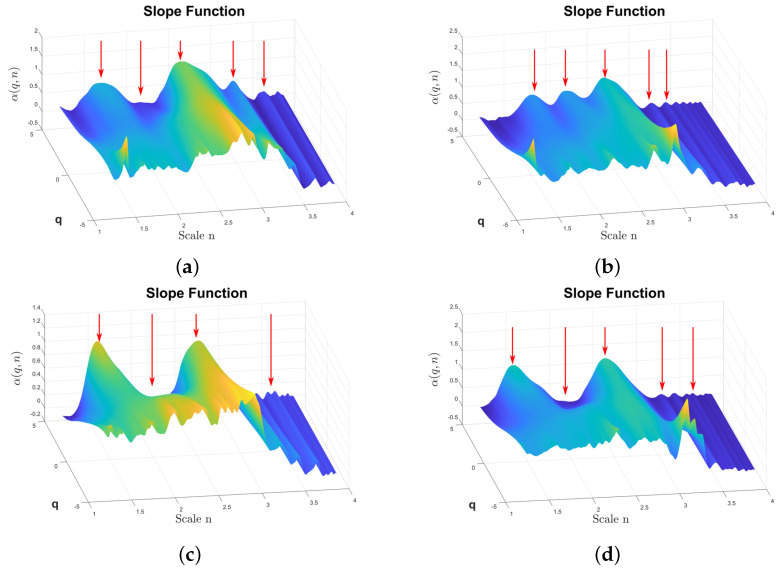
α(q,n) from the reciprocating compressor multi-fault dataset calculated by DFA1. The α amplitude is color coded, the highest amplitudes are plotted using light yellow, the lower amplitudes are plotted in dark blue. The red arrows indicate amplitude features that highlights differences between fault classes. (**a**) α(q,n) from the class F1, (**b**) α(q,n) from the class F6, (**c**) α(q,n) from the class F10, (**d**) α(q,n) from the class F13.

In particular, the application of MFDFA corresponding to DFA1 to the signals shown in Figure 8 allows one to obtain the fluctuation $F_{q}\left(n\right)$ as an array of size 11×257 that can be shown as a surface in Figure 9 for classes F1, F6, F10, and F13 of the multi-fault vibration signal dataset. The analysis was performed considering a signal fragment of 32,768 time samples. Even when there are differences between the surfaces $F_{q}\left(n\right)$ shown, they are difficult to grasp from such surfaces. A better representation that emphasizes the rapid changes in the slope of the surfaces in Figure 9 corresponds to the slope surface α(q,n) shown in Figure 10. In this case, the differences between the surfaces corresponding to F1, F6, F10, and F13 can be observed. In particular, for large positive values of *q*, the differences in the surface are shown using red arrows. The surface has two prominent peaks on the left, with the amplitude varying depending on the fault type. In fault type F6, a third peak appears between the previously mentioned peaks. On the right side of the surface, there are also differences between the fault types shown in this figure.

**Figure 11:**
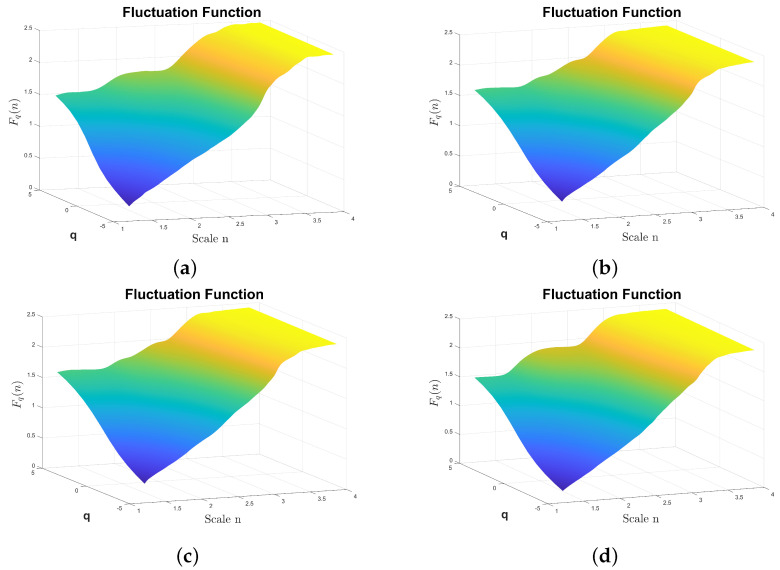
Fq(n) from the reciprocating compressor multi-fault dataset calculated by DFA2. The fluctuation amplitude is color coded, the highest amplitudes are plotted using light yellow, the lower amplitudes are plotted in dark blue. (**a**) Fq(n) from the class F1, (**b**) Fq(n) from the class F6, (**c**) Fq(n) from the class F10, (**d**) Fq(n) from the class F13.

**Figure 12:**
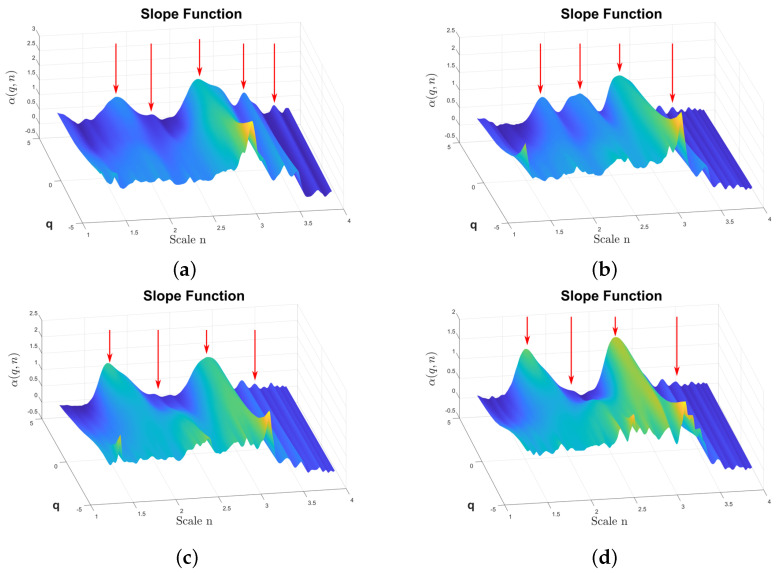
α(q,n) from the reciprocating compressor multi-fault dataset calculated by DFA2. The α amplitude is color coded, the highest amplitudes are plotted using light yellow, the lower amplitudes are plotted in dark blue. The red arrows indicate amplitude features that highlights differences between fault classes. (**a**) α(q,n) from the class F1, (**b**) α(q,n) from the class F6, (**c**) α(q,n) from the class F10, (**d**) α(q,n) from the class F13.

**Figure 13:**
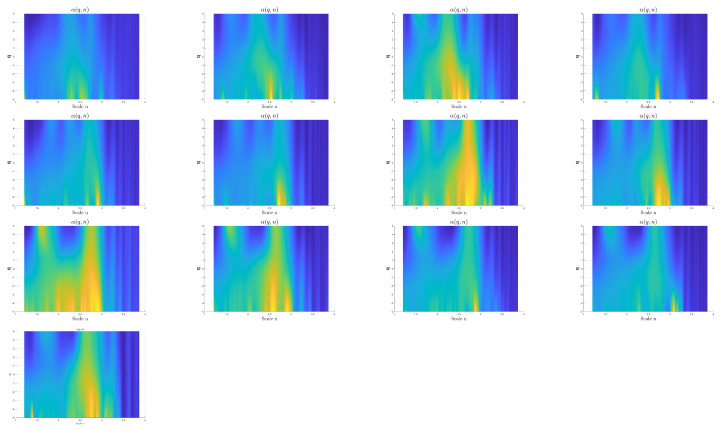
α(q,n) calculated from $F_{q}\left(n\right)$ using DFA1. The α amplitude is color coded, the highest amplitudes are plotted using light yellow, the lower amplitudes are plotted in dark blue. The upper rows show α(q,n) for signals extracted from faults F2 to F13. The healthy case F1 is represented by the α(q,n) located in the bottom row.

The MSDFA also facilitates the calculation of DFA2, which involves fitting a polynomial of degree two. The results are shown in Figure 11, corresponding to the fluctuation surfaces $F_{q}\left(n\right)$ calculated for classes F1, F6, F10, and F13. Additionally, the surfaces corresponding to the slope α(q,n) are shown in Figure 12where class differences are observed more clearly. This figure shows the differences between classes using red arrows, particularly for large positive values of q. On the left, the three arrows show a valley and two peaks with amplitudes that change for the different fault types. The two arrows at the right of the plot also show peaks that change in amplitude. A representation of the surface α(q,n) for vibration signals extracted from all classes in the multi-fault dataset is shown in Figure 13. The feature array α(q,n) is shown as a 2D image with low amplitude values in dark blue and higher amplitude values in light yellow. The figure shows the surface for classes F2 to F13 representing faulty classes in the upper three rows. The surface α(q,n) for the healthy class F1 is shown in the bottom row. There are differences in all the surfaces representing each of the classes considered. In addition, this feature array can be converted to images that can be used as input for deep learning models for fault classification. In particular, we use the CNN model for fault classification.

### 5.1. Classification Results for the Centrifugal Pump

Results concerning the cross-validation of several classical machine learning models and the CNN model, trained with the MFDFA features extracted from the vibration signal dataset for the centrifugal pump, are shown in Table 4. The dataset includes 13 different conditions, and the cross-validation was performed for DFA1 and DFA2 features. In both cases, the accuracy attained for all machine learning models is higher than 99%. In both cases, the random forest models corresponding to the subspace discriminant attained the highest accuracy, corresponding to 100%. Similarly, the sensitivity, specificity, and $F_{1}$ -score are all higher than 99% for all machine learning models. Even when the CNN model attained a classification accuracy higher than 99%, the accuracy attained was slightly lower than that of the RFSD model.

As the cross-validation process was repeated ten times for the classical models and five times for the CNN model, we calculated the accuracy variation during the cross-validation experiment. The average accuracy, the standard deviation, the minimum, and the maximum are reported in Table 5 for both DFA1 and DFA2. In both cases of DFA1 and DFA2, the highest accuracy and lower standard deviation are attained by the random forest model RFSD, corresponding to an average accuracy of 100% with a standard deviation of 0.00%. The accuracy attained by the CNN model is 99.73% for DFA2 with a standard deviation of 0.27%. In this case, the minimum is 99.39%, and the maximum is 100%.

**Figure 14:**
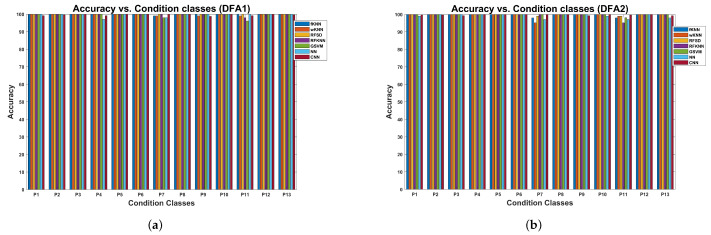
Cross-validation accuracy as a function of the fault class. (**a**) The features correspond to the DFA1 extracted from the centrifugal pump multi-fault vibration signals; (**b**) the features correspond to the DFA2 extracted from the centrifugal pump multi-fault vibration signal dataset.

The results of the cross-validation procedure, in terms of accuracy for each of the conditions, are shown in Figure 14 for the models trained with the DFA1 (a) and DFA2 (b) features. Concerning DFA1, classes P4, P7, P9, and P11 (see Table 1) are classified with lower accuracy by some models compared with the maximum of 100%. Regarding the DFA2 features, the challenging classes were P7 and P11. In particular, the wKNN model attained an accuracy of 95.2% for class P7, and the RFKNN model attained the same accuracy as class P11. Other models also had lower accuracy concerning these classes. However, most of the classes were classified with high accuracy with all the models tested.

**Figure 15:**
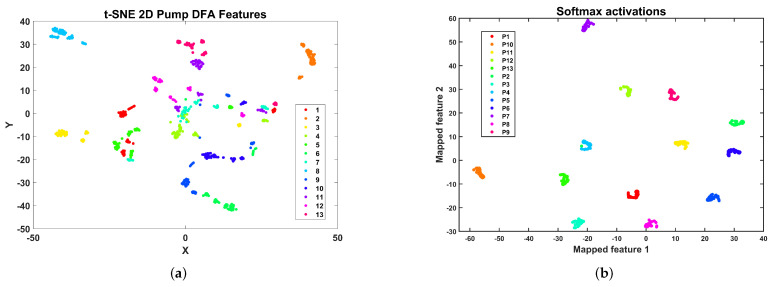
t-SNE representation of the feature space and the activations of the softmax layer. (**a**) t-SNE 2D representation of the DFA feature space for the centrifugal pump multi-fault vibration signal dataset, (**b**) the activations correspond to the softmax layer in the CNN architecture.

The t-distribution stochastic neighbor embedding (t-SNE) [62] enables the construction of a qualitative visual representation of the cluster structure of the DFA features dataset. The t-SNE allows one to visualize high-dimensional data by constructing a mapping from the original space to a lower 2D or 3D space map. The representation of the DFA features as a 2D space map is shown in Figure 15a. In this representation, each of the classes is composed of several clusters. However, the machine learning models tested can accurately classify all the centrifugal pump conditions. The small clusters in the feature space could be related to the six experimental conditions regulating pressure in the discharge valve. In Figure 15b, the 2D t-SNE plot for the activations in the softmax layer of the CNN architecture is shown. This plot shows 13 clusters corresponding to the classes considered in the centrifugal pump multi-fault dataset. This fact shows that the selected CNN architecture efficiently classifies this set of faults with high precision.

### 5.2. Classification Results for the Reciprocating Compressor

#### 5.2.1. Results for the Multi-Faults Vibration Dataset

Results for the cross-validation procedure for the reciprocating compressor using the multi-fault vibration signal dataset are presented in Table 6. The random forests with sub-space discriminant achieved 95.38% accuracy for the feature set DFA1, and the CNN achieved 97.78% accuracy for the DFA2 features, both having the highest classification accuracy. The accuracy difference compared to the other evaluated machine learning models is significant. The CNN model ranked second in terms of classification accuracy, achieving 88.63% for DFA1. The RFSD model took second place with 97.44% for the DFA2 features. In all models, the specificity reached values higher than 98.12%. The lowest FPR is 0.19%, corresponding to the CNN model trained with DFA2 features, and the highest is 1.89% for the weighted kNN model trained with DFA1.

The variation in accuracy during the cross-validation experiment is presented in Table 7 for the machine learning models trained with DFA1 and DFA2 features. The lowest standard deviation of 3.15% is attained with the RFSD model trained with DFA1 features. Similarly, in the case of DFA2 features, the lowest standard deviation of 2.09% is also attained by the RFSD model, although the CNN model attains the highest accuracy. However, this model had a standard deviation of 2.33%, which is slightly higher than the standard deviation attained by the RFSD model.

**Figure 16:**
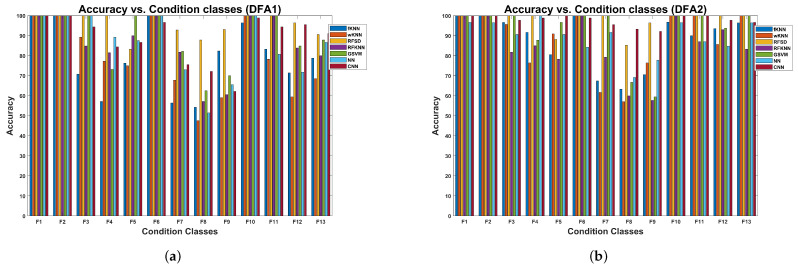
Cross-validation accuracy as a function of the fault class. (**a**) The features correspond to the DFA1 extracted from the reciprocating compressor multi-fault vibration signals. (**b**) The features correspond to the DFA2 extracted from the reciprocating compressor multi-fault vibration signal dataset.

The results concerning the variation in accuracy as a function of the condition class are shown in Figure 16a for models trained with DFA1 features. There is a set of classes where several models exhibit lower classification accuracies due to the nature of the fault. The first group corresponds to classes F3, F4, and F5, all with inner race cracks (see Table 3). The second group comprises classes F7, F8, and F9, all with roller element cracks. The last group comprises classes F11, F12, and F13, all with outer race cracks. In these groups, the common valve faults are corrosion, fracture, and broken spring. In contrast, fault conditions involving valve seat wear exhibit higher classification accuracy. The faults are heavily weighted by the fault related to the valve rather than the fault related to the bearing. In this plot, the RFSD model has clear advantages concerning the accuracy attained.

The results for the models trained with DFA2 features are shown in Figure 16b. In this case, we can observe the same group of faults where the models attain low accuracy. The CNN and RFSD models have advantages concerning classification accuracy compared to the rest.

**Figure 17:**
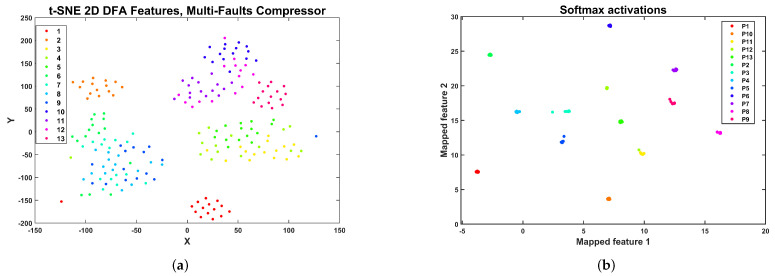
t-SNE representation of the feature space and the activations of the softmax layer. (**a**) t-SNE 2D representation of the DFA feature space for the reciprocating compressor multi-fault vibration signal dataset; (**b**) the activations correspond to the softmax layer.

A 2D representation of the DFA2 feature space for the compressor multi-fault dataset using t-SNE is shown in Figure 17a. In this representation, there are five large clusters: a cluster with points of the healthy class F1 (at the bottom), a cluster for the F2 condition (top left), a cluster with samples from conditions F10, F11, F12, and F13 (top right), a cluster with samples from conditions F6, F7, F8, and F9 (middle left), and a cluster composed of samples from conditions F3, F4, and F5 (middle right). Recalling the information in Table 3, it seems like the roller element crack and outer race crack define the two most significant clusters located in the diagonal feature representation, and the valve faults are subsumed into each of the clusters, making it challenging to attain an accurate classification. A 2D t-SNE representation of the activations in the softmax layer of the CNN architecture trained with DFA2 features is shown in Figure 17b. The model can transform the features’ original representation into 13 well-separated clusters, enabling accurate classification.

#### 5.2.2. Results for the Multi-Valve Vibration Dataset

The cross-validation results using the reciprocating compressor, the multi-valve fault vibration dataset, are presented in Table 8. The cross-validation was performed for both DFA1 and DFA2 feature sets. The random forests with sub-space discriminant models for the DFA1 features attain the highest classification accuracy of 99.61%. In contrast, for the DFA2 features, the highest classification accuracy of 99.80% is attained with the CNN model. In the case of the models trained with DFA1, the CNN attained the second-best accuracy, corresponding to 97.58%. In contrast, with the models trained with DFA2, the RFSD and SVM attained the second-highest accuracy of 99.02%, and the NN attained the third-best accuracy of 98.04%. Concerning the models trained with DFA1, the lowest accuracy is attained by random forests with sub-space KNN, with a value of 91.76%. The kNN model achieved the lowest accuracy of 93.92% when trained with DFA2 features. The values attained by the specificity range between 99.25% and 99.98%. The FPR ranges between 0.01% and 0.75%.

The accuracy variation during the cross-validation experiment is presented in Table 9. Concerning the models trained with DFA1 features, the lowest standard deviation of 1.24% is attained with the RFSD model, followed by the CNN model with 1.45%. The lowest standard deviation of 0.18% is attained with the CNN model trained with DFA2 features. The RFSD and GSVM models with 1.03% follow this result.

**Figure 18:**
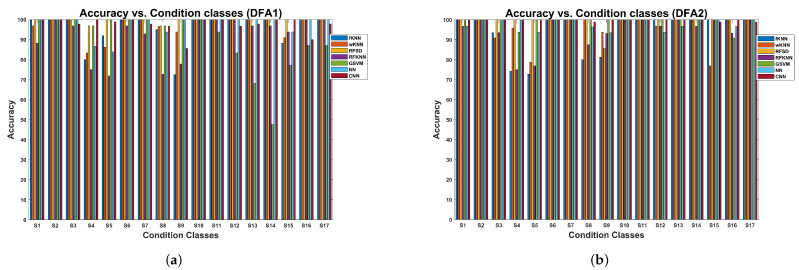
Cross-validation accuracy as a function of the fault class. (**a**) The features correspond to the DFA1 extracted from the multi-valve vibration signals of the reciprocating compressor; (**b**) the features correspond to the DFA2 extracted from the multi-valve vibration signal dataset of the reciprocating compressor.

The variation in accuracy as a function of the condition class for models trained with DFA1 features, extracted from the multi-valve fault dataset of the reciprocating compressor, is shown in Figure 18a. Several machine learning models have difficulties classifying several condition classes. In particular, S4, S5, S8, S9, S14, and S15 have lower accuracy for several machine learning models. However, the RFSD model provides the highest accuracy for most of the classes. Concerning the type of fault (see Table 2), the dominant faults are the valve plate fracture and broken spring. The results of the accuracy variation for models trained with DFA2 are shown in Figure 18b. The classes where several classical machine learning models have lower accuracy are S3, S4, and S5. The second group comprises classes S8 and S9; the last group corresponds to S12, S15, and S16. In these groups, the valve plate fracture appears in several instances. Concerning this dataset, the CNN and RFSD models provide the highest accuracy for most of the classes.

**Figure 19:**
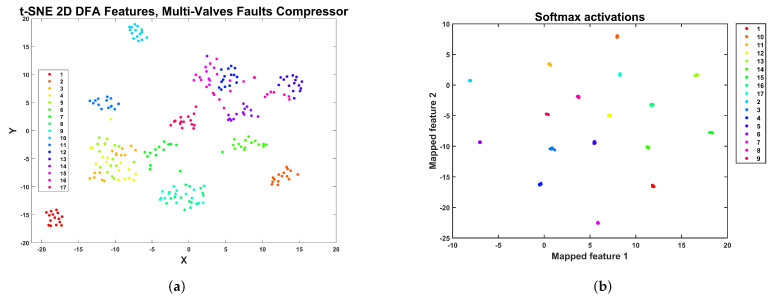
t-SNE representation of the feature space and the activations of the softmax layer. (**a**) t-SNE 2D representation of the DFA2 feature space for the multi-valve vibration signal dataset of the reciprocating compressor; (**b**) the activations correspond to the softmax layer of the CNN architecture.

The t-SNE 2D representation of the feature space is shown in Figure 19a. In this representation, several conditions form an isolated cluster. In particular, we can observe isolated clusters for the healthy condition S1 and faulty conditions, S2, S6, S10, S11, and S17. A large cluster is composed of S12, S13, S14, S15, and S16. Another cluster is composed of S3, S4, S5, and S7. Finally, there is a cluster comprising faults S8 and S9. In this case, it is difficult to draw any conclusion concerning the cluster structure and the nature of the valve faults. However, the CNN and RFSD machine learning models can accurately classify all the valve faults. Concerning the CNN model, the 2D t-SNE representation for the activations in the softmax layer of the CNN architecture is shown in Figure 19b. In this case, the model has transformed the cluster structure of the DFA2 features dataset of the multi-valve dataset of the reciprocating compressor, as shown, where 17 clusters represent the 17 health classes of the dataset. This cluster structure is amenable to highly accurate classification.

## 6. Discussion

Previous research performed in our research group has been reported concerning the proposal of several features useful for fault diagnosis in centrifugal pumps and reciprocating compressors. Research reported in [49] investigated the proposal of several feature sets useful for fault classification using classical machine learning models. Specifically, the research compared a statistical feature set composed of 12 features (*Statistical*), an information entropy feature set composed of 15 features (*InfoEntropy*), a non-linear entropy feature set composed of 14 features (*Entropy*), and the concatenation of all features previously mentioned, composed by 41 features *Allfeat*. In addition, the comparison also included the 2D spectrogram of the vibration signal fed to a CNN model for fault classification. The research used the same experimental test rig. The investigation was validated using the centrifugal pump dataset with 13 fault conditions and the multi-valve fault dataset acquired from the reciprocating compressor. A performance comparison is reported in Table 10. The percentage accuracy attained with the scale-fractal DFA representation used with the CNN model is higher than that attained with the spectrogram feature representation of the vibration signal combined with a CNN model. In addition, the accuracy attained by the α(q,n) features is also higher than the accuracy attained by the rest of the statistical and non-linear entropy-based features investigated in [49] for the reciprocating compressor. Concerning the centrifugal pump, the accuracy attained by the α(q,n) features is slightly lower than the accuracy attained by the combination of statistical and non-linear entropy-based features (*Allfeat*). Although the accuracy provided by the feature set focused on non-linear entropy features is high, a limitation of such features, like approximate entropy, is that their estimation requires calculating distances of *n*-dimensional signal vectors representing the phase space for the non-linear dynamical system. The length of these vectors *n* should be large enough to represent the dynamical system accurately. However, increasing *n* has an impact on the computational cost. In contrast, even when the MFDFA also requires the calculation of distances between the $N_{n}$ segments and the local trend of the signal, the length of these segments is lower than the length of the vectors used for approximate entropy calculation, and the computational cost is lower for MFDFA. This computational efficiency is a relevant advantage of the MFDFA method proposed in this document.

Research reported in [63] investigated the usefulness of Poincaré images generated from vibration signals, combined with CNN models for fault classification in a reciprocating compressor. The research was validated using the multi-valve dataset with 17 valve faults from the reciprocating compressor. Poincaré images represent a promising 2D representation of time series, such as the vibration signal that could be combined with deep learning models for fault classification. However, further research is still necessary to improve the classification accuracy and the range of possible applications. The highest accuracy attained with Poincaré images was 94.97%, which is lower than the accuracy attained with the proposed scale-fractal DFA feature representation.

Two additional feature types were reported in [31], helping to classify faults in a reciprocating compressor using classical machine learning models. The results concerning the multi-fault dataset, including 13 conditions, were classified with an accuracy of 100% considering symbolic dynamic features and 99.4% with the complex correlation measure when such features were combined with random forest classifiers. This accuracy is higher than that attained with the scale-fractal DFA representation combined with CNN, which corresponds to 97.78%. Concerning the multi-valve fault dataset, the symbolic dynamics combined with random forest classifiers attained an accuracy of 100%, and the complex correlation measure achieved an accuracy of 91.7%. In contrast, the scale-fractal DFA representation combined with CNN attained a classification accuracy of 99.80% for the multi-valve fault dataset. Although the symbolic dynamic features and complex correlation measure achieved high accuracy in the reciprocating compressor, these features have not been investigated in centrifugal pumps. Moreover, these features are intended mainly for use in combination with classical machine learning models.

Considering the previous comparison with other features proposed in our research group, the scale-fractal DFA feature representation is highly accurate. It has advantages in classification performance compared to other types of features. In addition, their bidimensional representation can be efficiently connected to deep learning classifiers such as the CNN model. Vibration signals acquired from centrifugal pumps and reciprocating compressors exhibit inherent non-stationary and non-linear dynamics derived from the superimposed contributions of multiple fault modes and interacting internal components. Furthermore, their dynamic characteristics fluctuate in response to such external driving forces under various operating conditions involving speed or load variations. The confluence of non-linear effects arising from fault interactions, compounded by behavioral variability due to changes in the operating regime, generates complexities that make it difficult to effectively extract characteristics indicative of fault frequency using conventional techniques [64]. A relevant advantage of the scale-fractal DFA feature type is that this feature set can be extracted directly from the vibration signal without the need to apply denoising methodologies. The proposed feature type is highly robust as it can provide high classification accuracy by estimating the features directly from the raw vibration signal.

At this stage, the main goal of this research is to propose a feature extracted from vibration signals that could be useful for fault classification in centrifugal pumps and reciprocating compressors. Their implementation in a real-time application requires further research to optimize the computation time required for the feature extraction. Specifically, the feature extraction method runs on a laptop computer with an Intel Pentium i7-6700HQ CPU 2.60 GHz and 12 GB of RAM using Matlab 2021a. It takes 92.0 s to process a vibration signal to extract $\alpha_{1}\left(q,n\right)$ and $\alpha_{2}\left(q,n\right)$ . Once an RF model is trained, it takes only 0.09 s to classify such a signal. Overall, the fault classification approach with the proposed features could be implemented in real-time using carefully selected hardware and optimized parameters according to the specific application.

The fractal-scale feature representation we are proposing is robust because it is extracted from raw vibration signals and provides high classification accuracy with either CNN or classical machine learning models. The algorithm for calculating the features uses maximally overlapping boxes that provide robustness to the estimation, as shown in Figure 2. This robustness is a relevant advantage concerning other methods proposed in the literature that estimate the DFA features after applying complex preprocessing methodologies. However, we acknowledge that further research into this type of feature is needed to assess its potential usefulness in other rotating machines for anomaly detection and estimating the remaining useful life of rotating machines. Furthermore, evaluating how features and classification methods behave in the presence of noisy signals and missing data is necessary. A comparison with other time-frequency representations also represents a topic of investigation for this type of feature.

## 7. Conclusions

The MFDFA features enable accurate fault classification in both a centrifugal pump and a reciprocating compressor. In particular, the slope of the fluctuation provides a 2D feature representation suitable for classifying faults using deep learning models such as CNN. In addition, this type of feature can be used with classical machine learning models for fault classification.

The multi-scale calculation of the detrended fluctuation analysis using maximally overlapped blocks allows for the estimation of the slope of fluctuation with minimal noise. These DFA features are useful for classifying faults in rotating machinery, such as centrifugal pumps and reciprocating compressors. A relevant advantage of the scale-fractal DFA feature representation is their robustness, enabling feature extraction from vibration signals without requiring denoising preprocessing.

The methodology for fault classification was validated using a vibration signal dataset representing 13 different multi-fault conditions in the centrifugal pump. Cross-validation was conducted using various machine learning models, and the accuracy attained in all tested models was higher than 99%. The method was also tested using two vibration signal datasets from a reciprocating compressor. The first dataset corresponds to 13 multi-faults that combine faults from the second-stage valve and the bearings. The CNN model attained the highest classification accuracy, corresponding to 97.78%. The second vibration signal dataset corresponds to 17 multi-valve fault conditions, and the highest classification accuracy was attained by the CNN model, corresponding to 99.80%.

The CNN model demonstrated a low standard deviation in the accuracy measure for most experiments during cross-validation.

The comparison between DFA1 and DFA2 features showed similar results for the centrifugal pump. However, in the reciprocating compressor, clear advantages in accuracy were observed between DFA1 and DFA2, particularly with DFA2 allowing for the highest accuracy using the CNN model.

Further research into this type of feature is necessary to assess their potential usefulness in other rotating machines, for detecting anomalies, and for predicting the remaining useful life of these machines. Comparing these features to other time-frequency representations is also an important research subject, as is their application to other signals recorded for condition monitoring.

## Figures and Tables

**Table 1:** Fault combinations in valves of the centrifugal pump. The healthy state is denoted as HTH . The numbers in the columns for stages 5 to 10 represent the severity of the faulty condition presented in the second column.

Fault Label	Impeller Fault	Stage 10	Stage 9	Stage 8	Stage 7	Stage 6	Stage 5	Rest of Stages
P1	HTH	HTH	HTH	HTH	HTH	HTH	HTH	HTH
P2	PEB	2	1	HTH	HTH	HTH	HTH	HTH
P3	PEB	5	4	3	2	1	HTH	HTH
P4	PEB	8	7	6	5	4	3	HTH
P5	POB	2	1	HTH	HTH	HTH	HTH	HTH
P6	POB	5	4	3	2	1	HTH	HTH
P7	POB	8	7	6	5	4	3	HTH
P8	ICB	1	HTH	HTH	HTH	HTH	HTH	HTH
P9	ICB	4	3	2	1	HTH	HTH	HTH
P10	ICB	6	5	4	3	2	1	HTH
P11	IB	1	HTH	HTH	HTH	HTH	HTH	HTH
P12	IB	4	3	2	1	HTH	HTH	HTH
P13	IB	6	5	4	3	2	1	HTH

**Table 2:** The reciprocating compressor was configured with the following set of valve faults. The corrosion and fracture are located in the valve plate. The first column lists the stage where the fault is found (1 or 2) and the type of valve (intake valve or discharge valve). Faults S14–S17 are located in the intake valve *2* of the first stage.

Stage and Valve Type	Fault Type	Fault Label
All stages	HTH	S1
2, DV	VSW	S2
2, DV	VPC	S3
2, DV	VPF	S4
2, DV	BS	S5
2,IV	VSW	S6
2, IV	VPC	S7
2, IV	VPF	S8
2, IV	BS	S9
1, DV	VSW	S10
1, DV	VPC	S11
1, DV	VPF	S12
1, DV	BS	S13
1, IV 2	VSW	S14
1, IV 2	VPC	S15
1, IV 2	VPF	S16
1, IV 2	BS	S17

**Table 3:** Multi-faults in the reciprocating compressor involve combining faults in the valves and roller bearings. For the fault type, the corrosion and fracture are located in the valve plate. The valve exhibiting the seeded fault is in the second stage’s discharge valve, located close to the fan pulley.

Label	Valve, 2S–DV	Bearings, B1
F1	Healthy	Healthy
F2	Valve seat wear	IRC
F3	Corrosion	IRC
F4	Fracture	IRC
F5	Broken Spring	IRC
F6	Valve seat wear	REC
F7	Corrosion	REC
F8	Fracture	REC
F9	Broken Spring	REC
F10	Valve seat wear	ORC
F11	Corrosion	ORC
F12	Fracture	ORC
F13	Broken Spring	ORC

**Table 4:** Performance metrics for DFA1 and DFA2 obtained during cross-validation of several machine learning models for the centrifugal pump dataset. The best performance is presented with bold numbers. In both experiments reported, the RFSD model attained the best performance concerning the metrics reported.

Features	Model	Accuracy	Sensitivity	Specificity	FPR	$F_{1}$ -Score
DFA1	kNN	99.85	99.85	99.99	0.01	99.85
	wKNN	99.85	99.85	99.99	0.01	99.85
	RFSD	**100.00**	**100.00**	**100.00**	**0.00**	**100.00**
	RFKNN	99.92	99.92	99.99	0.00	99.92
	GSVM	99.69	99.69	99.97	0.03	99.69
	NN	99.62	99.62	99.97	0.03	99.61
	CNN	99.67	99.67	99.97	0.03	99.67
DFA2	kNN	99.62	99.62	99.97	0.03	99.62
	wKNN	99.62	99.63	99.97	0.03	99.62
	RFSD	**100.00**	**100.00**	**100.00**	**0.00**	**100.00**
	RFKNN	99.77	99.77	99.98	0.02	99.77
	GSVM	99.92	99.92	99.99	0.00	99.92
	NN	99.08	99.09	99.92	0.08	99.08
	CNN	99.73	99.73	99.98	0.03	99.73

**Table 5:** Cross-validation performance for both DFA1 and DFA2 for the centrifugal pump. The metrics represent the average accuracy, standard deviation, and maximum and minimum obtained during the k-fold cross-validation. The best performance is presented with bold numbers. In both experiments reported, the RFSD model attained the best performance concerning the metrics reported.

Features	Model	Mean	Std	Min	Max
DFA1	kNN	99.85	0.24	99.23	100
	wKNN	99.85	0.37	99.23	100
	RFSD	**100.00**	**0.00**	**100.00**	**100**
	RFKNN	99.92	0.32	99.23	100
	GSVM	99.69	0.40	99.23	100
	NN	99.62	0.54	98.46	100
	CNN	99.67	0.44	98.77	100
DFA2	kNN	99.62	0.40	99.23	100
	wKNN	99.62	0.40	99.23	100
	RFSD	**100.00**	**0.00**	**100.00**	**100**
	RFKNN	99.77	0.54	98.46	100
	GSVM	99.92	0.32	99.23	100
	NN	99.08	0.73	97.69	100
	CNN	99.73	0.27	99.39	100

**Table 6:** Performance metrics for DFA1 and DFA2 obtained during the cross-validation of several machine learning models for the reciprocating compressor multi-fault dataset. The best performance is presented with bold numbers. In the experiments reported, the RFSD and CNN models attained the best performance concerning the metrics reported.

Features	Model	Accuracy	Sensitivity	Specificity	FPR	$F_{1}$ -Score
DFA1	kNN	77.95	79.02	98.17	1.83	77.70
	wKNN	77.18	78.65	98.12	1.89	77.18
	RFSD	**95.38**	**95.71**	**99.62**	**0.38**	**95.36**
	RFKNN	81.03	81.48	98.42	1.58	81.08
	GSVM	88.21	89.27	99.02	0.98	88.34
	NN	84.10	85.09	98.68	1.32	84.33
	CNN	88.63	88.63	99.05	0.95	89.64
DFA2	kNN	87.94	88.23	99.01	0.99	87.42
	wKNN	86.67	88.01	98.90	1.10	86.56
	RFSD	97.44	97.69	99.79	0.21	97.45
	RFKNN	84.87	85.06	98.75	1.26	84.60
	GSVM	91.79	92.64	99.32	0.68	91.99
	NN	88.71	89.41	99.06	0.94	88.73
	CNN	**97.78**	**97.78**	**99.81**	**0.19**	**97.78**

**Table 7:** Cross-validation performance for DFA1 and DFA2 for the multi-fault dataset of the reciprocating compressor. The metrics represent the average accuracy, standard deviation, and maximum and minimum obtained during the k-fold cross-validation.The best performance is presented with bold numbers.

	Algorithm	Mean	Std	min	max
MFDFA1	kNN	77.95	5.30	71.79	87.18
	wKNN	77.18	7.30	64.10	87.18
	RFSD	**95.38**	**3.15**	**89.74**	**100.00**
	RFKNN	81.03	4.55	74.36	89.74
	GSVM	88.21	5.01	74.36	92.31
	NN	84.10	6.14	74.36	92.31
	CNN	88.63	3.49	86.75	94.87
MFDFA2	kNN	87.95	2.43	84.62	92.31
	wKNN	86.67	3.15	82.05	92.31
	RFSD	97.44	**2.09**	**94.87**	**100.00**
	RFKNN	84.87	4.90	76.92	92.31
	GSVM	91.79	3.38	84.62	97.44
	NN	88.72	5.16	79.49	94.87
	CNN	**97.78**	2.33	94.02	99.57

**Table 8:** Performance metrics for DFA1 and DFA2 obtained during cross-validation of several machine learning models for the multi-valve dataset of the reciprocating compressor. The best performance is presented with bold numbers. In both experiments reported, the RFSD and CNN models attained the best performance concerning the metrics reported.

Features	Model	Accuracy	Sensitivity	Specificity	FPR	$F_{1}$ -Score
DFA1	kNN	95.10	95.75	99.70	0.30	95.01
	wKNN	96.86	96.91	99.80	0.20	96.85
	RFSD	**99.61**	**99.62**	**99.97**	**0.02**	**99.61**
	RFKNN	91.76	91.72	99.49	0.51	91.61
	GSVM	87.25	87.21	99.25	0.75	87.20
	NN	97.45	97.53	99.84	0.16	97.46
	CNN	97.58	97.58	99.85	0.15	97.57
DFA2	kNN	93.92	94.22	99.62	0.38	93.96
	wKNN	95.49	95.66	99.72	0.28	95.43
	RFSD	99.02	99.16	99.94	0.06	99.01
	RFKNN	94.71	94.69	99.67	0.33	94.65
	GSVM	99.02	99.10	99.94	0.06	99.02
	NN	98.04	98.18	99.88	0.12	98.03
	CNN	**99.80**	**99.80**	**99.99**	**0.01**	**99.80**

**Table 9:** Cross-validation performance for DFA1 and DFA2 for the multi-valve dataset of the reciprocating compressor. The metrics represent the average accuracy, standard deviation, and maximum and minimum obtained during the k-fold cross-validation. The best performance is presented with bold numbers. In both experiments reported, the RFSD and CNN models attained the best performance concerning the metrics reported.

	Algorithm	Mean	Std	min	max
MFDFA1	kNN	95.10	2.96	90.20	100.00
	wKNN	96.86	2.11	94.12	100.00
	RFSD	**99.61**	**1.24**	**96.08**	**100.00**
	RFKNN	91.76	3.18	88.24	98.04
	GSVM	87.25	1.39	84.31	88.24
	NN	97.45	2.45	94.12	100.00
	CNN	97.58	1.45	95.75	99.35
MFDFA2	kNN	93.92	3.51	88.24	100.00
	wKNN	95.49	2.08	92.16	98.04
	RFSD	99.02	1.03	98.04	100.00
	RFKNN	94.71	2.93	88.24	98.04
	GSVM	99.02	1.03	98.04	100.00
	NN	98.04	2.07	94.12	100.00
	CNN	**99.80**	**0.18**	**99.67**	**100.00**

**Table 10:** Comparison of the performances of several features extracted from the reciprocating compressor (RC) and the centrifugal pump (CP) obtained during the 10-fold cross-validation of classical and CNN models. The accuracies are represented in percentage values. The dataset for RC denotes the multi-valves with 17 fault conditions, and for CP, the dataset denotes the multi-faults with 13 fault conditions. The best performance is presented with bold numbers.

Machine	Accuracy *Statistical* +SVM [49]	Accuracy *InfoEntropy* +SVM [49]	Accuracy *Entropy* +SVM [49]	Accuracy *AllFeat* +SVM [49]	Accuracy α(q,n) +CNN	Accuracy Spectrogram +CNN [49]
CP	71.03	96.32	97.30	**99.81**	99.73	98.61
RC	86.91	96.83	97.57	97.90	**99.80**	96.74

## Data Availability

The data used in this research are available upon reasonable request to the corresponding authors.

## Abbreviations

The following abbreviations are used in this manuscript:

BS	Broken Spring
CBR	Convolutional, Batch normalization, and ReLU layer
CNN	Convolutional neural networks
CP	Centrifugal Pump
D	Dropout layer
DFA	Detrended fluctuation analysis
DFA2	Detrended fluctuation analysis with quadratic approximation
DV	Discharge valve
EDM	Electrical Discharge Machining
EWT	Empirical Wavelet Transform
FC	Full connected layer
GSVM	Support vector machine with Gaussian kernel
GSVM	Gaussian support vector machines
HTH	Healthy
IB	Imbalance impeller
ICB	Impeller channel blockage
IMF	Intrinsic mode function
IRC	Inner race crack
IV	Inlet valve
kNN	k-Nearest Neighbors
MFDFA	Multi-fractal detrended fluctuation analysis
MP	Max-pooling layer
MSB	Modulation signal bispectrum
NN	Neural network
ORC	Outer race crack
PCA	Principal component analysis
PEB	Pitting at the entrance of the impeller blades
POB	Pitting at the output of the impeller blades
RC	Reciprocating Compressor
REC	Roller element crack
ReLU	Rectified linear unit
RF	Random Forests
RFKNN	Random forest subspace k-nearest neighbor
RFKNN	Random forest subspace kNN
RFSD	Random forest subspace discriminant
ROC	Receiver operator curve
SMF	State-adaptive morphological filter
SOFT	Softmax layer
SVM	Support vector machine
VMD	Variational mode decomposition
VPC	Valve plate corrosion
VPF	Valve plate fracture
VSW	Valve seat wear
wKNN	Weighted k-nearest neighbor

## Appendix A. Tables and Figures

The appendix includes several tables and figures that complement the topic reported in the paper.

### Appendix A.1. Tables

The tables report the environmental and experimental conditions used during the vibration signal acquisition.

**Table A1:** Values of the environmental parameters required for operating the reciprocating compressor and the centrifugal pump. These parameters must be measured at least one meter around the compressor.

Parameter	Units	Limit	Value
Room temperature	°C	Min	14
Room temperature	°C	Max	24
Relative Humidity	Percent	Min	42%
Relative Humidity	Percent	Max	60%
Environmental noise before starting compressor	dBA	Max	48
Environmental noise during experiment	dBA	Max	80

**Table A2:** Experimental conditions for the centrifugal pump.

Experimental Conditions	Description	Discharge Pressure (bar)
C1	Regulated discharge valve up to 5.5 bar	5.5±0.2
C2	Regulated discharge valve up to 6.5 bar	6.5±0.2
C3	Regulated discharge valve up to 7.5 bar	7.5±0.2
C4	Regulated discharge valve up to 8.5 bar	8.5±0.2
C5	Regulated discharge valve up to 9.5 bar	9.5±0.2
C6	Regulated discharge valve up to 10.4 bar	10.4±0.2

**Table A3:** Values of the physical parameters of the compressor during acquisition.

Parameter	Units	First Stage	Second Stage	Tank
Tank pressure	bar	-	-	2.9 to 3.1
Inlet pressure	bar	−0.1 to 0.1	Variable	-
Discharge pressure	bar	1.3 to 2.5	2.9 to 4.1	-
Surface temperature	°C	37 to 65	38 to 72	-
in the intake valve cover				
Surface temperature	°C	42 to 50	40 to 62	-
in the discharge valve cover				

### Appendix A.2. Figures

A set of figures that describes the centrifugal pump and the reciprocating compressor are included in this appendix.
